# Toxicological Assessment of Trace Element Exposure in Relation to Sudden Unexplained Death (SUD): Environmental Geochemistry and Dietary Risk in Central-Eastern Yunnan, China

**DOI:** 10.3390/toxics13121078

**Published:** 2025-12-14

**Authors:** Yangchun Han, Litao Hao, Shixi Zhang, Kunli Luo

**Affiliations:** 1 School of Geosciences and Surveying Engineering, China University of Mining and Technology Beijing, Beijing 100083, China; 2 Institute of Geographic Sciences and Natural Resources Research, Chinese Academy of Sciences, Beijing 100101, China; 3 Office of the State-Owned Assets Committee, Hebei Normal University, Shijiazhuang 050024, China

**Keywords:** toxic trace elements, environmental toxicology, sudden unexplained death (SUD), dietary exposure, hazard index

## Abstract

Sudden Unexplained Death (SUD) has been reported in specific regions of Yunnan Province, China, yet its environmental causes remain unclear. This study aimed to explore the potential toxicological link between trace element exposure and SUD by investigating the concentrations of multiple elements in soil, corn, and drinking water from typical SUD and non-SUD villages in central-eastern Yunnan. Elemental abundances were determined, and dietary exposure and non-carcinogenic health risks for adults and children were assessed. Results showed that soils in SUD villages were markedly deficient in Na but enriched in Se and Cr compared with non-SUD villages. Corn and drinking water were generally low in essential trace elements, with notable Co deficiency in corn and Fe, Li, Mn, and Cu deficiency in water. Cr and Mn in corn were identified as the main contributors to non-carcinogenic risks, especially for children. Comparative analysis with Keshan Disease (KD) villages in Shaanxi Province indicated distinct elemental patterns, suggesting different pathogenic mechanisms. Overall, environmental Na deficiency and exposure to Cr and Mn may be potential toxicological factors associated with SUD, warranting further investigation into their physiological effects and regional disease etiology.

## 1. Introduction

Sudden Unexplained Death (SUD) in Yunnan Province is a summer-aggregated lethal disease [1]. Its clinical manifestations primarily include symptoms such as fatigue, syncope, coma, and diarrhea, though most victims die suddenly without exhibiting any obvious symptoms [2]. SUD in Yunnan Province occurs in impoverished mountainous or semi-mountainous areas of central and western Yunnan, China, at elevations ranging from 1800 m to 2400 m. Since the late 1970s, this disease has been prevalent in 8 cities (prefectures) and 22 counties (districts) in the central and northwestern regions of Yunnan Province, with over 300 reported cases of sudden death [3,4]. It exhibits distinct spatiotemporal clustering, rapid onset, and high fatality rates, along with familial aggregation characteristics [5,6]. Affected individuals were predominantly farmers, with fatalities occurring mainly among young and middle-aged females, though cases were also observed in children and the elderly [7].

However, the etiology of SUD in Yunnan Province remains unclear. Currently, four main hypotheses have been proposed: viral infection (e.g., Coxsackievirus), deficiency of trace elements (e.g., Se and Cr), topographical and geological factors (e.g., release of CO_2_ and CH_4_), and consumption of wild poisonous mushrooms (e.g., Trogia venenata) [1,2,3,5]. Many scholars believe that there is a strong association between the consumption of Trogia venenata and SUD in Yunnan Province [1,3,8]. However, some studies have documented cases in the same village where sudden deaths occurred both among those who consumed the mushroom and those who did not, with relatively clear evidence for both scenarios [3,9]. Therefore, the SUD in Yunnan remains a serious endemic public health problem with an unknown etiology.

To investigate the etiology of SUD in Yunnan Province, studies have found that the symptoms and environmental background of SUD are highly similar to those of Keshan Disease (KD), which is prevalent in China. First, both diseases present symptoms such as fatigue and syncope [2,10]. Second, patients affected by both diseases reside in impoverished mountainous areas far from urban centers, primarily leading self-sufficient lifestyles with limited access to external food sources [1,10]. Finally, the affected areas of both diseases exhibit significant spatiotemporal clustering in their spatial distribution, with diseased villages often surrounded by several non-diseased villages, and the distance between diseased and non-diseased villages being very close [8,11].

The deficiency of the trace element Se is considered closely associated with KD [12]. This is because the endemic areas of KD geographically overlap significantly with low-Se regions, and Se supplementation in these areas has been shown to effectively reduce the incidence of KD [13]. This has led to the hypothesis that SUD in Yunnan Province may also be related to environmental trace elements. Therefore, similar to studies on the environmental geochemical background of Keshan disease, investigating the elemental characteristics of different environmental media (soil, crops, and drinking water) in SUD areas at a micro-scale (comparing affected and unaffected villages) and understanding the differences in elemental abundance and dietary risks for residents in these areas are not only crucial for revealing the environmental geochemical features of SUD regions but also provide important references for further research into the pathogenic mechanisms of the disease.

Of course, it is important to note that the symptomatic manifestations of SUD and KD are not entirely identical. Specifically, patients with SUD in Yunnan Province often die suddenly without exhibiting any obvious prior symptoms [2]. Therefore, comparing the differences in the content of trace elements such as Se in various environmental media (soil, crops, and drinking water) between SUD villages in Yunnan Province and KD villages in Shaanxi Province is also of great significance.

Therefore, this study focused on the Honghe Prefecture and Chuxiong Prefecture in the central-eastern part of Yunnan Province as the research areas, and the element concentrations in the soil, grain, and drinking water of typical diseased villages and adjacent non-diseased villages were measured, respectively. On this basis, a further comparative analysis was conducted to compare the differences in the contents of elements such as Se in different environmental media between the SUD villages in Yunnan Province and the KD villages in Shaanxi Province, to preliminarily analyze whether SUD is also related to the environmental geochemical background. The specific research contents include analyzing and comparing the element concentrations in the soil, crop, and drinking water between typical SUD villages and adjacent non-SUD villages in Yunnan Province, as well as KD villages in Shaanxi Province, and assessing the risks of dietary food and drinking water for the residents in the typical SUD villages and non-SUD villages in the study area.

## 2. Materials and Methods

### 2.1. Study Area

**Figure 1:**
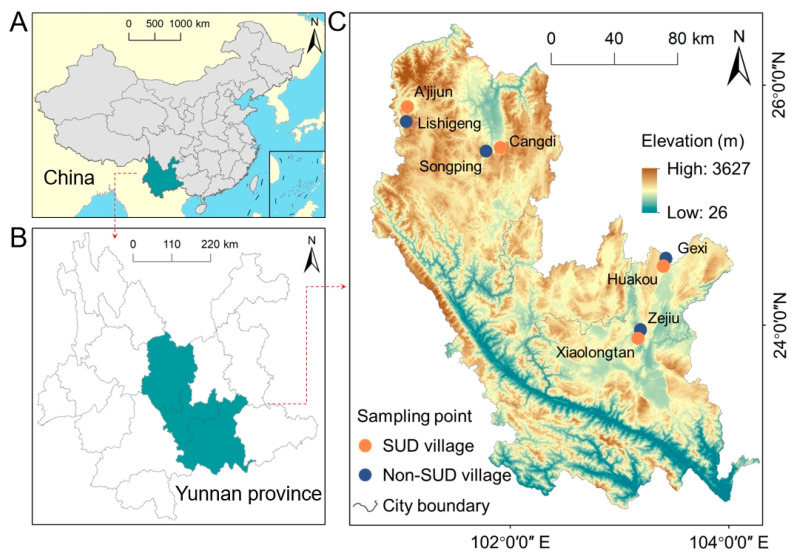
Location of study areas and distribution of sampling sites: (**A**) location of Yunnan Province; (**B**) location of the study area in Yunan Province; (**C**) location map of the sampling sites.

The study area for the SUD in Yunnan Province includes Honghe Prefecture (Mile City and Kaiyuan City) and Chuxiong Prefecture (Dayao County and Lufeng City) (Figure 1) [14]. The study area is located in the southeastern part of Yunnan Province, with an altitude range of 26 m to 3627 m. The terrain is mainly composed of high mountains and deep valleys. This region has a subtropical monsoon climate, with the rainy season from May to October accounting for more than 80% of the total annual rainfall [15]. The affected areas are predominantly impoverished mountainous and semi-mountainous regions, distant from urban centers and with limited transportation access. Due to poor soil quality, crop yields are low, with maize being the primary staple grown in these mountainous areas, resulting in a generally low standard of living [14]. Residents have a simple dietary structure, relying mainly on purchased rice and self-grown maize. Their intake of animal protein and legumes is low, vegetable varieties are limited, and drinking water is sourced from mountain spring water [2].

### 2.2. Sample Collection and Processing

Since the cases of SUD in Yunnan Province occurred in specific rural households (showing a “point-like” distribution), environmental samples were collected exclusively from diseased households in the affected villages to ensure research accuracy and representativeness, while samples from non-diseased households in neighboring villages were collected as controls. Therefore, this study adopted villages as the sampling unit (Table 1). All SUD environmental samples were obtained from households with documented SUD cases and their crop cultivation areas, whereas non-SUD environmental samples were collected from households in adjacent villages with no history of SUD cases. Moreover, given the similarity in the distribution pattern of KD cases and SUD incidents in Yunnan Province, the same environmental sampling methodology was applied.

In summary, the research team collected a total of 24 cultivated soil samples, 18 natural drinking water samples (no water samples were collected from Huakou village and Xiaolongtan village due to drought conditions), and 24 corn samples from four diseased/non-diseased villages in the SUD area of Yunnan Province in 2023. For comparison, corresponding soil (18 samples), corn (18 samples), and natural drinking water (18 samples) were collected from six typical diseased villages in the KD area of Shaanxi Province (Table 1). It should be noted that to ensure the representativeness of the samples, each sample type was collected in triplicate at every sampling site. The collection and processing of samples followed the same methods as reported in the literature [16,17,18,19]. Briefly, soil samples were air-dried and ground to 200 mesh for analysis; corn samples were rinsed with deionized water, dried in an oven at 60 °C, and then pulverized to 100 mesh using a high-speed universal grinder for analysis; natural drinking water samples were stored in pre-cleaned polyethylene bottles and preserved at 4 °C for analysis.

**Table 1:** Information on the sampling site [14,20].

Area	City	County	Diseased Villages	Coordinates (E°, N°)	Non-Diseased Villages	Coordinates (E°, N°)
SUD area	Chuxiong	Dayao	A’jijun	(101.059°, 25.823°)	Lishigeng	(101.042°, 25.701°)
SUD area	Chuxiong	Lufeng	Cangdi	(101.900°, 25.400°)	Songping	(101.786°, 25.383°)
SUD area	Honghe	Mile	Huakou	(103.429°, 24.482°)	Gexi	(103.434°, 24.565°)
SUD area	Honghe	Kaiyuang	Xiaolongtan	(103.146°, 23.793°)	Zejiu	(103.161°, 23.887°)
KD area	Tongchuan	Yijun	Xingshi	(109.309°, 35.459°)	-	-
KD area	Xianyang	Yongshou	Tianyun	(108.019°, 34.851°)	-	-
KD area	Xianyang	Xunyi	Yangjiahutong	(108.595°, 35.293°)	-	-
KD area	Xianyang	Xunyi	Zhangjiabian	(108.632°, 35.324°)	-	-
KD area	Xi’an	Lantian	Jinshan	(109.380°, 34.287°)	-	-
KD area	Shangluo	Zhashui	Lijiagou	(109.540°, 33.718°)	-	-

Note: “-” no data.

### 2.3. Sample Analysis and Quality Control

Determination of major and trace elements in soil samples: Firstly, approximately 0.05 g of soil sample was weighed, and 20 mL of mixed acid (HNO_3_:HF:HClO_4_ = 5:5:1, *v*:*v*:*v*) was added. The mixture was heated at 180 °C ± 10 °C until the solution became transparent. Then, the transparent solution was further heated to remove excess acid until no white acid fumes were observed. Finally, after cooling, the remaining solution was diluted to 25 mL with ultrapure water. Major and trace elements were determined using ICP-OES (PerkinElmer, Optima 5300DV, Waltham, MA, USA, LOD: 1 μg/kg) and ICP-MS (PerkinElmer, Elan DRC-e, Waltham, MA, USA, LOD: 0.001 μg/kg), respectively. Determination of Se and As in soil samples: Firstly, approximately 0.05 g of soil sample was weighed, and 20 mL of mixed acid (HNO_3_:HClO_4_ = 5:1, *v*:*v*) was added. The mixture was heated at 180 °C ± 10 °C until the solution became transparent. Then, the transparent solution was further heated to remove excess acid until no white acid fumes were observed. Finally, after the solution cools to room temperature, 5 mL of HCl (6 mol/L) was added, and the solution was heated again until white acid fumes appeared. After cooling again, 1 mL of HCl (12 mol/L) was added, and the solution was diluted to 15 mL with ultrapure water for the detection of Se. For As detection, 3 mL of the test solution was aliquoted, 1 mL of HCl (12 mol/L) and 1 mL of reducing agent (2.5% thiourea and 2.5% ascorbic acid) were added, the mixture was allowed to stand for 30 min, and then it was diluted to 15 mL. Hydride generation atomic fluorescence spectrometry was used to determine Se and As (HG-AFS, Beijing Haiguang Instruments Co., Ltd., Beijing, China, LOD: 0.01 μg/kg) [17,19].

Determination of major and trace element concentrations in corn samples: Firstly, approximately 0.5 g of the corn sample was weighed, and 10 mL of mixed acid (HNO_3_:HClO_4_ = 5:1, *v*:*v*) was added. The mixture was heated at 180 °C ± 10 °C until the solution became transparent. Then, the transparent solution was further heated to remove excess acid until no white acid fumes were observed. Finally, after cooling, the remaining solution was diluted to 15 mL with ultrapure water. Major and trace elements were determined using ICP-OES (PerkinElmer, Optima 5300DV, Waltham, MA, USA, LOD: 1 μg/kg) and ICP-MS (PerkinElmer, Elan DRC-e, Waltham, MA, USA, LOD: 0.001 μg/kg), respectively. Determination of Se and As concentrations in corn samples: Firstly, approximately 0.5 g of corn sample was weighed, and 10 mL of mixed acid (HNO_3_:HClO_4_ = 9:1, *v*:*v*) was added. The mixture was heated at 180 °C ± 10 °C until the solution became transparent. Then, the transparent solution was further heated to remove excess acid until no white acid fumes were observed. Finally, after the solution cooled to room temperature, 5 mL of HCl (6 mol/L) was added, and the solution was heated again until white acid fumes appeared. After cooling again, 1 mL of HCl (12 mol/L) was added, and the solution was diluted to 15 mL with ultrapure water for the detection of Se. Add 1 mL of HCl (12 mol/L) and 1 mL of reducing agent (2.5% thiourea and 2.5% ascorbic acid) to the Se detection residual solution, let it stand for 30 min, and dilute it to 15 mL for As detection. Use hydride generation atomic fluorescence spectrometry to determine Se and As (HG-AFS, Beijing Haiguang Instruments Co., Ltd., Beijing, China, LOD: 0.01 μg/kg) [18,19].

Determination of element concentrations in natural drinking water samples: The concentrations of major and trace elements were determined using ICP-OES (PerkinElmer, Optima 5300DV, Waltham, MA, USA, LOD: 1 μg/kg) and ICP-MS (PerkinElmer, Elan DRC-e, Waltham, MA, USA, LOD: 0.001 μg/kg), respectively. Among them, tandem ICP-MS (ICP-MS/MS, 8900, Agilent, Palo Alto, CA, USA) was used with O_2_ as the reaction gas to analyze Se and As in water under MS/MS mode. The operating parameters for ICP-OES, ICP-MS, ICP-MS/MS, and HG-AFS are listed in Tables S1, S2, S3, and S4, respectively [17]. HNO_3_, HF, HClO_4_, and HCl were all purchased from Chemical Reagent Research Institute Co., Ltd., Beijing, China.

### 2.4. Health Risk Assessment Model

The Hazard Index (HI) proposed by the United States Environmental Protection Agency (USEPA) has been widely and successfully applied to assess the potential non-carcinogenic risks to human health caused by trace elements through skin, inhalation, and oral ingestion, etc. [21,22]. Since oral ingestion is the primary route for the human body to absorb trace elements from crops and water, the USEPA’s proposed health risk assessment model was utilized to evaluate the health risks to humans (including adults and children) caused by consuming drinking water and crops [22,23]. It should be noted that this study investigated the maximum health risk of trace elements for residents under a specific dietary pattern (single diet). Although this approach simplifies the actual situation (the diversity of diets), it can assess the maximum safe health risk of corn. The ratio of the chronic daily intake (CDI) to the standard reference dose (RfD) calculated by the theoretical model (hazard quotient—HQ) is used as the evaluation criterion. The HI is used to calculate the sum of the hazard quotients of all trace elements exposed through each pathway. When HI > 1, it indicates that the consuming population may have a health risk from the exposure to trace elements caused by consuming drinking water-crops; when HI ≤ 1, it indicates that the consuming group of drinking water-crops does not have a health risk from the exposure to trace elements. The smaller the HI, the smaller the exposure risk [22]. Table 2 lists the parameters and definitions of the health risk assessment model. Specifically, it is calculated by Formulas (1)–(3):

(1) $$CDI=\frac{(C_{corn}\times {IR}_{corn}+C_{water}\times {IR}_{water})\times EF\times ED}{BW\times AT}$$

(2) $$HQ=\frac{CDI}{RfD}$$

(3) HI=∑HQs

### 2.5. Statistic Analysis

This study mainly utilized ArcGIS 10.2, Origin 2021, SPSS 22.0 and Excel 2016 for chart drawing and data analysis. Specifically, ArcGIS 10.2 was used to create a location map of SUD areas and distribution map of sampling sites; Origin 2021 was employed to draw bar charts; SPSS 22.0 was utilized to conduct one-way analysis of variance (ANOVA) and independent sample *t*-tests on the element concentrations in soil, crops and drinking water in the SUD area in Yunnan Province (A *p*-value < 0.05 was considered statistically significant.); and Excel 2016 was used for the creation of three-line tables, etc.

The quality of soil samples was controlled using the national standard substance (GBW07403, IGGE, Langfang, China) and the quality of corn samples was controlled using the national standard substance (GBW10012, IGGE, Langfang, China) [18,19]. The linear regression correlation coefficient (R^2^) of the calibration curve exceeded 0.999. Each batch of samples included two duplicate samples and two blank samples. Re-analysis was performed every 15 samples to ensure that the relative error between the two results remained within ±10% [16,19]. Each sample was measured three times, and the reported data represent the average of the three measurements.

## 3. Results and Discussion

### 3.1. The Content of Se and Other Trace Elements in the Surface Soil

**Figure 2:**
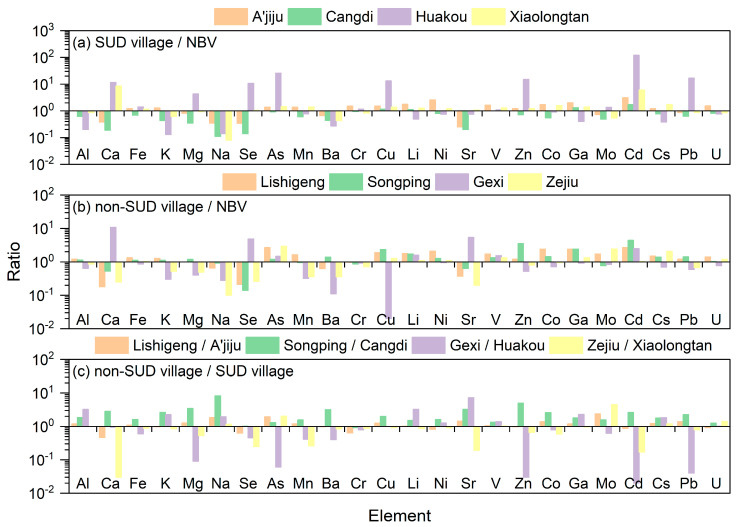
Ratio of element concentration in soil (NBV: national background value of soil).

The comparison results of soil element concentrations in the four SUD villages within the SUD area of Yunnan Province with the national background values of soil elements are presented in Figure 2a and Table 3. The study found that the concentrations of Cu, V, and Cd in the soil of the four SUD villages were all higher than the national background values of soil elements, while the concentrations of Na and Ba were all lower than the national background values [24]. Among them, the concentration of Cd in all the SUD villages was 1.70–121.63 times the national soil background value, followed by Cu (1.17–13.28 times). This might be due to the increase in human activities (such as mining, smelting, fertilizers, pesticides, and fuel combustion) over the past few decades, which led to the accumulation of these elements in the soil [25,26]. The concentrations of soil elements indicated by different letters in Table 3 showed significant differences among the four SUD villages (*p* < 0.05). One-way analysis of variance (ANOVA) revealed that Al, K and Cs showed significant differences across all SUD villages, while other elements exhibited significant variations between specific SUD villages (*p* < 0.05), indicating considerable overall heterogeneity. Among them, the coefficients of variation of all elements (except V) among the four SUD villages were all higher than 20%, and the coefficients of variation of Se, As, Cu, Zn, Cd and Pb exceeded 100%. This indicates that the element concentrations in the soil of the SUD villages vary significantly due to different geographical locations. Similar results can also be obtained from the maximum and minimum values of the same element in the four SUD villages, that is, the concentrations of Se, As, Cu, Zn, Cd and Pb in the four SUD villages can differ by more than ten times at most. To sum up, there are significant differences (e.g., Se, As, Cu, Zn, Cd and Pb) in soil element concentrations among different diseased villages in the SUD areas in Yunnan Province.

The comparison results of soil element concentrations in the four non-SUD villages within the SUD area of Yunnan Province with the national background values of soil elements are presented in Figure 2b and Table 4. The study found that the concentrations of As, Li, V, and Cd in the soil of the four non-SUD villages were all higher than the national background values of soil elements, while the concentrations of Na and Cr were all lower than the national background values [24]. Among them, the concentration of Cd in all the non-SUD villages was 1.02–4.44 times the national soil background value, followed by As (1.19–2.94 times). Similarly to the studies on SUD villages, this might be due to the increase in human activities (such as mining, smelting, fertilizer production, pesticide use, and fuel combustion) over the past few decades, which has led to the accumulation of these elements in the soil [25,26]. The concentrations of soil elements indicated by different letters in Table 4 show significant differences among the four non-SUD villages (*p* < 0.05). One-way analysis of variance (ANOVA) revealed that K, Na, Ba, Cu, Sr, and Zn showed significant differences across all non-SUD villages, while other elements exhibited significant variations between specific non-SUD villages (*p* < 0.05), indicating considerable overall heterogeneity. Among them, the coefficients of variation of all elements (except Fe, Cr, Li and V) among the four non-SUD villages were all higher than 20%, and the coefficients of variation of Ca, Se and Sr exceeded 100%. This indicates that the element concentrations in the soil of the non-SUD villages vary significantly due to different geographical locations. Similar results can also be obtained from the maximum and minimum values of the same element in the four non-SUD villages; that is, the concentrations of Ca, Se, Cu and Sr in the four non-SUD villages can differ by more than 26 times at most. To sum up, there are significant differences (e.g., Ca, Se, Cu and Sr) in soil element concentrations among different non-SUD villages in the SUD areas in Yunnan Province.

The *t*-test results of the average concentrations of soil elements in non-SUD and SUD villages in the SUD areas of Yunnan Province are listed in Table S5. It can be seen from Table S5 that there are significant differences in Al, Na, Cr, Cu, Li, V, Mo and Cd in the soil between SUD villages and non-SUD villages (*p* < 0.05). In addition, the comparison results of soil element concentrations between the four non-SUD villages and the SUD villages in the SUD area of Yunnan Province are presented in Figure 2c. The research found that the Cr concentration in the four SUD villages was higher than that in the non-SUD villages, while the concentrations of Al, Na, V and Cs in the four SUD villages were all lower than those in the non-SUD villages. In addition, the concentrations of Ca, Se, Ba, Zn and Cd in Songping village are higher than those in Cangdi village. The concentrations of Fe, Mg, As, Mn, Cu, Co and Pb in Lishigeng village and Songping village were higher than those in A’jiju village and Cangdi village, respectively. The concentrations of K and Ni in Songping village and Gexi village were higher than those in Cangdi village and Huakou village, respectively. However, the concentrations of Li, Sr and Ga in the Zejiu village were lower than those in Xiaolongtan village, the concentration of Mo in Gexi village was lower than that in Huakou village, and the concentration of U in Lishigeng village was lower than that in A’jiju village. It is noteworthy that the Se concentrations in A’jiju village (0.10 mg/kg), Huazhu village (3.11 mg/kg), and Xiaolongtan village (0.30 mg/kg) were all higher than those in the neighboring non-SUD villages (Lishigeng village: 0.06 mg/kg, Gexi village: 1.41 mg/kg, and Zejiu village: 0.08 mg/kg). Only Cangdi village (0.0416 mg/kg) had a slightly lower Se concentration than the neighboring non-SUD villages (Gexi village: 0.0419 mg/kg). Therefore, Se deficiency in soil is not a common characteristic in SUD villages and non-SUD villages in the SUD area of Yunnan Province. Instead, the soil concentrations of Al, Na, V, and Cs in SUD village were lower than those in non-SUD villages, and especially the deficiency of Na (lower than the national soil background value) is an important characteristic of the SUD villages in the SUD area of Yunnan Province.

### 3.2. The Content of Se and Other Trace Elements in Corn

**Figure 3:**
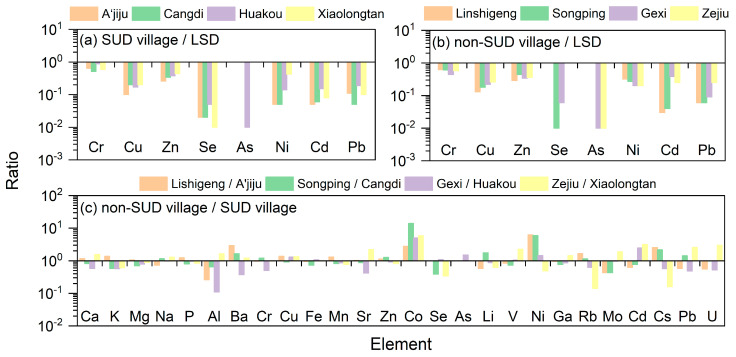
Ratio of element concentration in corn (LSD: Limit Standards of corn).

By comparing and analyzing the element concentrations of corn in four SUD villages with the food safety limit standards for elements (Figure 3a and Table 5), it was found that the concentrations of 8 elements (Cr, Cu, Zn, Se, As, Ni, Cd, and Pb) were all within the limit standards. Theoretically, these corns can be consumed by residents as safe agricultural products [27,28]. Among them, the concentrations of As and Se were far lower than the limit standards, ranging from 0.00 to 0.005 times and 0.014 to 0.054 times the limit standards, respectively. The concentrations of Cr and Zn were the closest to the limit standards, ranging from 0.51 to 0.88 and 0.26 to 0.44 times the limit standards, respectively. The element concentrations of corn indicated by different letters in Table 5 showed significant differences among the four SUD villages (*p* < 0.05). One-way analysis of variance (ANOVA) revealed that there were no significant differences in Ba and Sr among the four diseased villages in corn, while there were significant differences in K and P among the four diseased villages (*p* < 0.05). However, there were significant differences in other elements among the diseased villages (*p* < 0.05). Among these four SUD villages, the concentration differences of Cs and Rb were the greatest, with the maximum differences reaching 197.19 times and 31.11 times, respectively. Next were Ni (9.04 times) and Li (5.22 times), while the concentrations of other elements all had differences below 5 times.

By comparing and analyzing the element concentrations of corn in four non-SUD villages with the food safety limit standards for elements (Figure 3b and Table 6), it was also found that the concentrations of 8 elements (Cr, Cu, Zn, Se, As, Ni, Cd, and Pb) were all within the limit standards. Theoretically, these corns can also be consumed by residents as safe agricultural products [27,28]. Similarly to the research results of the SUD village, the concentrations of As and Se were far lower than the limit standards, ranging from 0.00 to 0.01 times and 0.00 to 0.06 times the limit standards, respectively. The concentrations of Cr and Zn were the closest to the limit standards, ranging from 0.44 to 0.62 and 0.29 to 0.44 times the limit standards, respectively. The element concentrations of corn indicated by different letters in Table 6 showed significant differences among the four non-SUD villages (*p* < 0.05). One-way analysis of variance (ANOVA) revealed that there were no significant differences in Cr among the four non-SUD villages in corn, while there were significant differences in K, Al, As and Mo among the four non-SUD villages (*p* < 0.05). However, there were significant differences in other elements among the non-SUD villages (*p* < 0.05). Among these four non-SUD villages, the concentration differences in Al were the greatest, with the maximum differences reaching 20.22 times, next were Cs (12.97 times) Cd (11.52 times) and Ba (7.41 times), while the concentrations of other elements all had differences below 5 times.

The *t*-test results of the average concentrations of corn elements in non-SUD and SUD villages in the SUD areas of Yunnan Province are listed in Table S6. It can be seen from Table S6 that there are significant differences in K, Mg, Al, Co, Se, As and Ni in the corn between SUD villages and non-SUD villages (*p* < 0.05). In addition, the comparison results of corn element concentrations between the four non-SUD villages and the SUD villages in the SUD area of Yunnan Province are presented in Figure 3c. The research found that the concentrations of Ca, K, Mg, P, Ba, Cu, Mn, Sr, Zn, Co, Ni, Rb and Cs in Lishigeng village were higher than those in A’jigu village. The concentrations of Na, Ba, Cr, Zn, Co, As, Li, Rb, Cs and Pb in Songping village are higher than those in Cangdi village. The concentrations of Cu, Fe, Co, Se, As, V, Ni and Cd in Gexi village are higher than those in Huakou village. The concentrations of Ca, Na, Al, Ba, Cu, Sr, Co, As, V, Ga, Mo, Cd, Pb and U in the Zejiu village were higher than those in Xiaolongtan village. The Ni concentration in Lishigeng village and A’jiju village differs by 6.27 times, while the differences in other elements are all less than 3 times. The Co and Ni in Songping village and Cangdi village differ by 14.06 times and 5.95 times, respectively, while the differences in other elements are less than 3 times. The concentrations of Co and Cd in Gexi village and Huakou village differed by 5.00 times and 2.48 times, respectively, while the concentrations of other elements differed by less than 2 times. The concentrations of Co, Cd and U in Zejiu village and Xiaolongtan village differed by 5.88 times, 3.15 times and 3.03 times, respectively. In summary, although the deficiency of Se in corn is a common feature in both SUD and non-SUD villages in the SUD area of Yunnan Province, the Se content in corn from SUD villages is higher than that from non-SUD villages. However, corn from SUD villages is more deficient in Co than that from non-SUD villages. Co is a metal component of vitamin B_12_, and Co deficiency leads to a reduction in the availability of B_12_, thereby increasing health problems related to B_12_ deficiency, such as anemia and nerve damage [29,30]. In addition, the concentrations of Co in the corn of the SUD village are also lower than those in some longevity regions (such as Jinxiang County in Shandong) and the Kaschin-Beck disease area (such as Yongshou County in Shaanxi) [19,31]. Therefore, the Co deficiency in corn from affected villages may pose additional health risks to local residents.

### 3.3. The Content of Se and Other Trace Elements in Water

**Figure 4:**
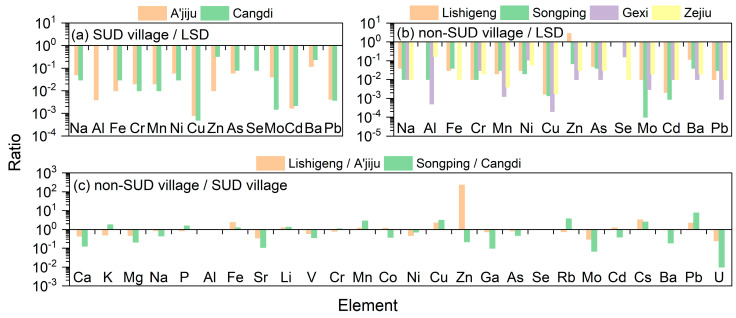
Ratio of element concentration in drinking water (LSD: limit standards of drinking water).

By comparing and analyzing the element concentrations in drinking water from two SUD villages (Huakou village and Xiaolongtan village, where no drinking water was collected) with the drinking water hygiene standards of China and the WHO (Figure 4a and Table 7), it was found that the concentrations of 14 elements (Na, Al, Fe, Cr, Mn, Ni, Cu, Zn, Se, As, Mo, Cd, Ba and Pb) were all within the limit standards. Theoretically, this drinking water can be consumed by residents as a safe product [32,33]. The element concentrations of drinking water indicated by different letters in Table 7 showed significant differences between the two SUD villages (*p* < 0.05). One-way analysis of variance (ANOVA) revealed that there were significant differences in Ca, K, Mg, Na, Al, Fe, Sr, Li, V, Mn, Ni, Cu, Zn, As, Se, Rb, Mo, Cs, Ba and U among the two SUD villages in drinking water, while there were no significant differences in other elements among the SUD villages (*p* < 0.05). Among these two SUD villages, the concentration differences in Zn and Mo were the greatest, with the maximum differences being 25.90 times and 25.46 times, respectively. These were followed by Sr, U, V, Cs, Ca, Fe, Mg, K, Ni and Ba, with maximum differences of 5.46, 3.62, 3.54, 3.30, 3.05, 2.71, 2.50, 2.36, 2.10 and 2.06 times, respectively. Then there were Na, Rb, Cu, Li, As, Mn, Cd, Co, Ga, Cr, P and Pb, with maximum differences of 1.85, 1.73, 1.71, 1.52, 1.44, 1.38, 1.27, 1.22, 1.21, 1.12, 1.11 and 1.10 times, respectively. However, the maximum difference among other elements is within one fold.

By comparing and analyzing the element concentrations in drinking water from four non-SUD villages with the drinking water hygiene standards of China and the WHO (Figure 4b and Table 8), it was found that the concentrations of Zn in Lishigeng village was exceeded the limit standards, while the concentrations of 14 elements (Na, Al, Fe, Cr, Mn, Ni, Cu, Zn, Se, As, Mo, Cd, Ba and Pb) in the other three non-SUD village were all within the limit standards [32,33]. Therefore, the drinking water in Songping village, Gexi village, and Zejiu village can theoretically be safely consumed by residents. The element concentrations of drinking water indicated by different letters in Table 8 showed significant differences among the four non-SUD villages (*p* < 0.05). One-way analysis of variance (ANOVA) revealed that there were significant differences in Cd, Ba and U among the four non-SUD villages in drinking water, while other elements exhibited significant differences between the non-SUD villages and non-SUD villages among the four villages (*p* < 0.05). Among these four non-SUD villages, the average concentration differences in Zn, U and Mo were the greatest, with the maximum differences being 319.67 times, 198.52 times and 172.56 times, respectively. These were followed by Pb, Li, Mn and Fe, with maximum differences of 30.47, 29.83, 25.43 and 22.20 times, respectively. Then there were Sr, Cd, Ca, Ga and Cu, with maximum differences of 17.17, 15.07, 13.86, 13.56 and 11.08 times, respectively. However, the maximum difference among other elements is within ten folds.

The *t*-test results of the average concentrations of drinking water elements in non-SUD and SUD villages in the SUD areas of Yunnan Province are listed in Table S7. It can be seen from Table S7 that there are significant differences in Ca, Mg, Mn, Ni, Cu, Ga, As, Cs, Ba, Pb and U in the drinking water between SUD villages and non-SUD villages (*p* < 0.05). In addition, the comparison results of drinking water element concentrations between the four non-SUD villages and the SUD villages in the SUD area of Yunnan Province are presented in Figure 4c. The research found that the concentrations of Fe, Li, Mn, Cu, Cs and Pb in the SUD villages were all lower than those in the non-SUD villages, while the concentrations of Ca, Mg, Na, Sr, V, Ni, Ga, As, Mo and U in the SUD villages were all higher than those in the non-SUD villages. In addition, the concentrations of Fe, Li, Mn, Co, Cu, Zn, Cd, Cs, Ba and Pb in Lishigeng village were higher than those in A’jiju village; the concentrations of K, P, Al, Fe, Li, Cr, Mn, Cu, Rb, Cs and Pb in Songping village were higher than those in Cangdi village. The concentrations of Zn and Cs in Lishigeng village and A’jiju village differ by 223.49 times and 3.21 times, respectively, while the differences in other concentrations are all less than 3 times. The concentrations of Pb, Rb and Cu in Songping village and Cangdi village differ by 7.44 times, 3.63 times and 3.09 times, respectively, and the differences in other element concentrations are all less than 3 times. Therefore, there was a deficiency of Se in drinking water in both the SUD and non-SUD villages in the SUD area of Yunnan Province. However, the drinking water in the SUD villages is more deficient in Fe, Li, Mn, Cu, Cs, and Pb compared to that in the non-SUD villages. Fe is a necessary nutrient for the human body and plays crucial biological functions, including blood production, oxygen transportation, energy metabolism, and immune processes [34]. Li promotes the development of the animal nervous system [35]. Mn is an essential trace component in human brain development, intracellular homeostasis, and the functions of various enzymes. Insufficient Mn intake can cause a series of health problems, including overall growth retardation, birth defects, decreased fertility, skeletal dysplasia, and disorders in lipid, protein, and carbohydrate metabolism [36,37]. Cu is a vital trace element for various physiological processes in the human body. Cu deficiency can lead to anemia, weakened immune function, delayed growth and development (especially in children), osteoporosis, abnormal cholesterol metabolism, and neurological disorders [38]. Since Cs and Pb are generally studied as harmful elements, their low concentrations in drinking water are beneficial to humans [39,40]. In addition, the concentrations of Fe, Li, Mn, and Cu in the drinking water of the SUD village are also lower than those in some longevity regions (such as Yangdong County in Guangdong, Jinxiang County in Shandong, and Jiangjin District in Chongqing) [31,41,42]. Therefore, the deficiency of Fe, Li, Mn and Cu in village drinking water may pose additional risks to human health.

### 3.4. Comparison of Element Content Between the Diseased Villages of SUD in Yunnan Province and KD in Shaanxi Province

**Figure 5:**
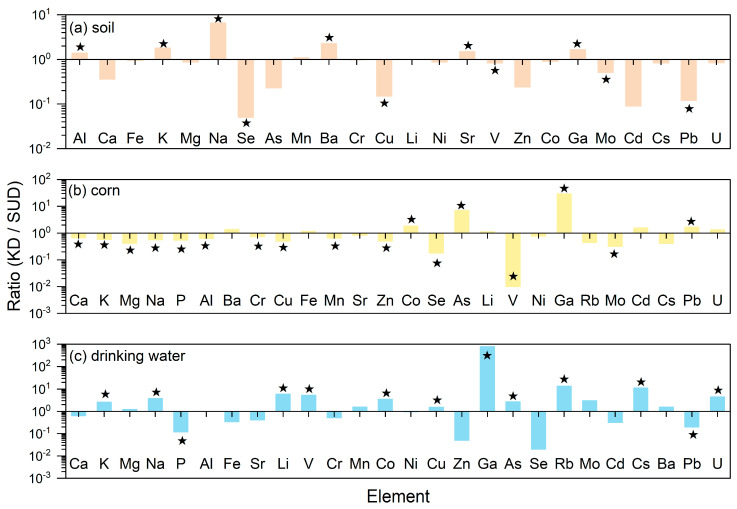
Comparison of the average elemental contents in soil, corn, and drinking water (★: *p* < 0.05).

The results of the *t*-test for the average concentrations of soil elements in the KD village of Shaanxi Province and the SUD village of Yunnan Province are listed in Table S8. As show in Table S8, there are significant differences (*p* < 0.05) in the soil elements of Al, K, Na, Se, Ba, Cu, Sr, V, Ga, Mo and Pb between the KD village and SUD village. In addition, the comparison results of the average concentrations of soil elements between the KD village in Shaanxi Province and the SUD village in Yunnan Province are listed in Figure 5a. The average concentrations of Al, K, Na, Mn, Ba, Li, Sr and Ga in the soil of the KD village are greater than those in the SUD village. Among them, the Na in the soil of the KD village is 6.47 times that of the SUD village, followed by Ba in the soil of the KD village, which is 2.27 times that of the SUD village, while Al, K, Mn, Li, Sr and Ga in the soil of the KD village are 1–2 times those of the SUD village. The average concentrations of Ca, Fe, Mg, Se, As, Cr, Cu, Ni, V, Zn, Co, Mo, Cd, Cs, Pb and U in the soil of the SUD villages of Yunnan Province were all higher than those in the KD villages of Shaanxi Province. Among them, the Se in the SUD villages in Yunnan Province was 21.90 times that of the KD villages in Shaanxi Province, followed by Cd, Pb and Cu in the soil of the SUD village, which were 11.00, 8.56 and 6.56 times higher than those in the KD villages of Shaanxi Province, respectively. Then As and Zn in the soil of the SUD village, which were 4.29 and 4.18 times higher than those in the KD villages of Shaanxi Province, respectively, while the Ca, Fe, Mg, Cr, Ni, V, Co, Mo, Cs and U in the SUD villages of Yunnan Province were 1–2 times higher than those in the KD villages of Shaanxi Province.

The results of the *t*-test for the average concentrations of corn elements in the KD village of Shaanxi Province and the SUD village of Yunnan Province are listed in Table S9. As shown in Table S9, there are significant differences (*p* < 0.05) in the corn elements of Ca, K, Mg, Na, P, Al, Cr, Cu, Mn, Zn, Co, Se, As, V, Ga, Mo and Pb between the KD village and SUD village. In addition, the comparison results of the average concentrations of corn elements between the KD village in Shaanxi Province and the SUD village in Yunnan Province are listed in Figure 5b. The average concentrations of Ba, Fe, Co, As, Li, Ga, Cd, Pb and U in the corn of the KD village are greater than those in the SUD village. Among them, the Ga in the corn of the KD village is 29.22 times that of the SUD village, followed by As in the corn of the KD village, which is 7.01 times that of the SUD village, while Ba, Fe, Co, Li, Cd, Pb and U in the corn of the KD village were 1–2 times those of the SUD village. The average concentrations of Ca, K, Mg, Na, P, Al, Cr, Cu, Mn, Sr, Zn, Se, V, Ni, Rb, Mo and Cs in the corn of the SUD villages of Yunnan Province were all higher than those in the KD villages of Shaanxi Province. Among them, the V in the SUD villages in Yunnan Province was 136.89 times that of the KD villages in Shaanxi Province, followed by Se, Mo, Cs, Mg and Rb in the corn of the SUD village, which were 5.61, 3.13, 2.44, 2.37 and 2.21 times higher than those in the KD villages of Shaanxi Province, respectively, while the Ca, K, Na, P, Al, Cr, Cu, Mn, Sr, Zn and Ni in the SUD villages of Yunnan Province were 1–2 times higher than those in the KD villages of Shaanxi Province.

The results of the *t*-test for the average element concentrations of drinking water in the KD village of Shaanxi Province and the SUD village of Yunnan Province are listed in Table S10. As shown in Table S10, there are significant differences (*p* < 0.05) in the drinking water elements of K, Na, P, Li, V, Co, Cu, Ga, As, Rb, Cs, Pb and U between the KD village and SUD village. In addition, the comparison results of the average concentrations of drinking water elements between the KD village in Shaanxi Province and the SUD village in Yunnan Province are listed in Figure 5c. The average concentrations of K, Mg, Na, Li, V, Mn, Co, Cu, Ga, As, Rb, Mo, Cs, Ba and U in the drinking water of the KD village are greater than those in the SUD village. Among them, the Ga in the drinking water of the KD village is 774.82 times that of the SUD village, followed by Rb, Cs, Li and V in the drinking water of the KD village, which is 13.40, 11.05, 5.95 and 5.22 times that of the SUD village, respectively. Then U, Na, Co, Mo, As and K in the drinking water of the SUD village, which were 4.40, 3.78, 3.46, 3.00, 2.66 and 2.56 times higher than those in the KD villages of Shaanxi Province, respectively, while Mg, Mn, Cu and Ba in the drinking water of the KD village were 1–2 times those of the SUD village. The average concentrations of Ca, P, Al, Fe, Sr, Cr, Ni, Zn, Se, Cd and Pb in the drinking water of the SUD villages of Yunnan Province were all higher than those in the KD villages of Shaanxi Province. Among them, the Se, Zn, P and Pb in the SUD villages in Yunnan Province was 62.05, 18.23, 8.57 and 5.06 times that of the KD villages in Shaanxi Province, respectively, followed by Cd, Fe and Sr in the drinking water of the SUD village, which were 3.08, 2.91 and 2.42 times higher than those in the KD villages of Shaanxi Province, respectively, while the Ca, Cr and Ni in the SUD villages of Yunnan Province were 1–2 times higher than those in the KD villages of Shaanxi Province.

To sum up, the average concentrations of Ca, Cr, Ni, Zn and Se in the soil, corn and drinking water of the SUD villages in Yunnan Province were all higher than those of the KD villages in Shaanxi Province. The concentrations of Ca, P, Al, Sr, Cr, Ni, Zn and Se in corn and drinking water, which are directly related to the residents’ diet, were higher in the SUD villages of Yunnan Province than in the KD villages of Shaanxi Province. The average concentrations of Ba, Li and Ga in the soil, corn and drinking water of the KD village in Shaanxi Province were all higher than those in the SUD village of Yunnan Province. The concentrations of Ba, Co, As, Li, Ga and U in corn and drinking water, which are directly related to the residents’ diet, were higher in the KD village of Shaanxi Province than in the SUD village of Yunnan Province. This indicates that the trace elements in the environmental medium of the KD Village in Shaanxi Province are more deficient than those in the SUD village of Yunnan Province, especially in corn and drinking water related to human life.

### 3.5. Health Risk Assessment of Residents in the SUD Village and Non-SUD Village of Yunnan Province

**Figure 6:**
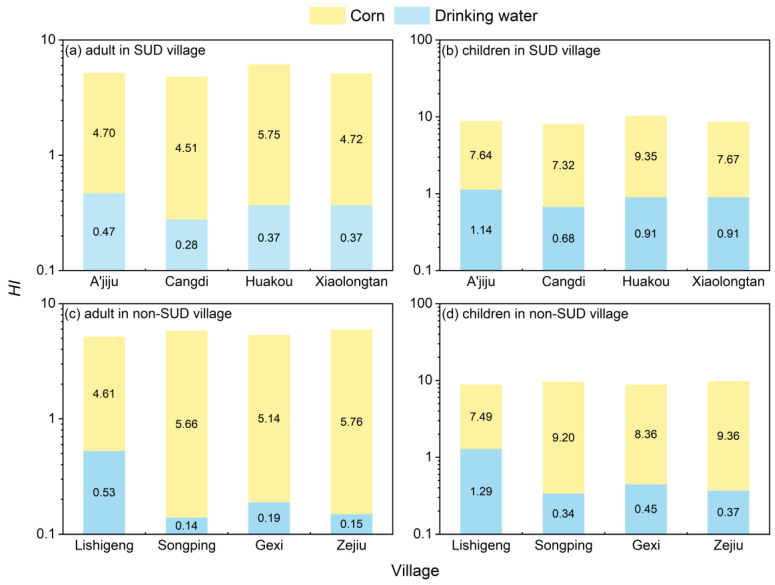
Trace element hazard index (HI) in corn—drinking water by adults and children.

The HI results for adults and children in the SUD villages of Yunnan Province through the ingestion of corn and drinking water are presented in Figure 6a and Figure 6b, respectively. The average HI for 17 trace elements in corn and drinking water consumed by adults in the four SUD villages was 5.29 (4.78–6.12), and for children it was 8.90 (8.00–10.26), both higher than the acceptable non-carcinogenic risk level of 1. Additionally, it was found in all four SUD villages that the non-carcinogenic risk for children was higher than that for adults. The non-carcinogenic risk for adults and children through the consumption of corn in the villages of A’jiju village, Cangdi village, Huakou village and Xiaolongtan village was higher than that through drinking water, and the contribution to the total hazard index was over 90% (for adults) and 87% (for children). Regarding the ingestion of the 17 trace elements via corn, Cr and Mn were the elements with the highest HQ values for both adults and children (Tables S11 and S12). The average HQ values for Cr and Mn in adults were 1.45 (1.12–1.95) and 1.17 (1.04–1.52), respectively, and for children they were 2.36 (1.82–2.31) and 1.91 (1.69–2.48), both higher than the acceptable non-carcinogenic risk level of 1, and contributing over 24.88% and 18.11%, respectively, to the total HQ. Regarding the ingestion of the 17 trace elements via drinking water, the HI and HQ of the 17 trace elements in drinking water for adults in the villages of A’jiju and Cangdi were both below 1, theoretically having no non-carcinogenic risk, and contributed over 77% to the total HQ. Since drinking water was not collected in Huakou village and Xiaolongtan village for health risk calculation, the drinking water concentration used for health risk calculation was replaced by the average value of drinking water from the other two villages. Therefore, these two villages were not discussed in this context. In summary, Cr and Mn in corn are the main non-carcinogenic risk factors in the SUD area of Yunnan Province.

Similarly, the HI results for adults and children in the non-SUD villages of Yunnan Province through the ingestion of corn and drinking water are presented in Figure 6c and Figure 6d, respectively. The average HI for 17 trace elements in corn and drinking water consumed by adults in the four non-SUD villages was 5.55 (5.14–5.91), and for children it was 9.22 (8.79–9.73), both also higher than the acceptable non-carcinogenic risk level of 1. Additionally, it was found in all four non-SUD villages that the non-carcinogenic risk for children was higher than that for adults. The non-carcinogenic risk for adults and children through the consumption of corn in the non-SUD villages was higher than that through drinking water, and the contribution to the total hazard index was over 89% (for adults) and 85% (for children). Regarding the ingestion of the 17 trace elements via corn (Table S13), Cr and Mn were the elements with the highest HQ values for both adults and children in Lishigeng village and Songping village. The average HQ values for Cr and Mn in adults from these two non-SUD villages were 1.37 and 1.33, respectively, while for children, they were 2.22 and 2.17, all exceeding the acceptable non-carcinogenic risk level of 1. The proportions all exceeded 23.00% and 22.00%, respectively. In Gexi village and Zejiu village, Cr and Mo were the elements with the highest HQ values for both adults and children, respectively. The average HQ values for Cr and Mo in adults from these two non-disease villages were 1.13 and 0.96, respectively, while for children, they were 1.84 and 1.56. These values also exceeded the acceptable non-carcinogenic risk level of 1, with the proportions exceeding this level surpassing 19.00% and 17.00%, respectively. Therefore, Cr, Mn and Mo in corn from non-SUD villages are the main non-carcinogenic risk factors. For adults, the HQ and HI values of 17 trace elements in drinking water are all below 1 (Table S14), and theoretically, there is no non-carcinogenic risk.

In conclusion, the non-carcinogenic risks for children in both the SUD villages and the non-SUD villages are higher than those for adults. This is mainly because children are more vulnerable to the threat of trace element pollution than adults [43]. The trace elements entering the human body through corn are more than those through drinking water, as the intake of crops is the main way for the human body to absorb trace elements [44]. In the SUD villages and non-SUD villages of Yunnan Province, the Cr and Mn in corn are the most significant non-carcinogenic risk factors, but the non-carcinogenic contribution of Mo in corn in the non-SUD villages (Gexi village and Zejiu village) is greater than that of Mn. The difference is that the non-carcinogenic risk contribution of consuming corn through corn for adults and children in non-SUD villages is much higher than that through drinking water. Moreover, all the drinking water in the SUD area has no non-carcinogenic risk and can be safely consumed.

## 4. Conclusions

This study investigated the elemental abundance in various environmental media and the associated dietary risks in typical diseased villages and adjacent non-diseased villages within the SUD area of central-eastern Yunnan Province. The key findings are as follows: The soil in the SUD villages did not show a Se deficiency phenomenon (the Se content in the SUD villages was 0.99–4.04 times that of the adjacent non-SUD villages) but was significantly deficient in Na (the Na content in the disease villages was only 16.7% of the national background value), and there were also significant differences in multielement concentrations between the SUD villages and the non-SUD villages. Therefore, the low Na in the soil may be an important environmental characteristic of the disease area. Although the eight limited elements (Cr, Cu, Zn, Se, As, Ni, Cd, and Pb) in the corn of the SUD area met the national safety standards, Cr (reaching 51–88% of the limit standard) constituted the main dietary risk source. The concentrations of various elements in drinking water were all within the safe threshold (except for Lishigeng village), but the drinking water in the SUD villages was significantly deficient in Fe, Li, Mn, Cu, Cs, and Pb (1.18–7.44 times lower than that in non-SUD villages). The KD villages in Shaanxi Province had significantly higher contents of Ba, Co, As, Li, Ga, and U in corn and drinking water than the SUD villages in Yunnan Province, reflecting essential differences in the pathogenic environmental factors between the two regions. This does not support the sharing of the same element deficiency mechanism between SUD in Yunnan Province and KD in Shaanxi Province. Corn consumption was the main non-carcinogenic risk pathway (accounting for >85%), with a slightly higher risk for children than adults. The Cr and Mn in the diseased areas (including SUD villages and non-SUD villages) constituted the core risk elements, while drinking water in adults had no health risks. This study provides a new perspective for the exploration of the environmental etiology of the SUD in Yunnan Province: low Na in the soil and Cr/Mn exposure in corn may be potential pathogenic factors, and the traditional theory of Se deficiency needs to be reexamined. The research results have important guiding significance for regional disease prevention and control and the safe regulation of crops.

## Figures and Tables

**Table 2:** Definition and reference value of some parameters for health risk assessment [22,23].

Factor	Definition	Adult	Children
C_corn_	Concentration of corn	- mg/kg	- mg/kg
C_water_	Concentration of water	- mg/kg	- mg/kg
IR_corn_	Ingestion rate of corn	0.450 kg/day	0.225 kg/day
IR_water_	Ingestion rate of water	2 L/day	1.5 L/day
EF	Exposure frequency	350 day/year	350 day/year
ED	Exposure duration	30 years	12 years
BW	Body weight	65 kg	20 kg
AT	Average time	10,950 days	4380 days
RfD	Reference dose	- mg/kg/day	- mg/kg/day
CDI	Chronic daily intake	- mg/kg/day	- mg/kg/day

“-”No data.

**Table 3:** Statistical results of soil element contents in disease villages of SUD areas in Yunnan Province.

Elements	Units	A’jiju	Cangdi	Huakou	Xiaolongtan	Mean	CV (%)	Max/Min	Background Value [24]
Al	%	6.72 ± 0.31 ^a^	4.07 ± 0.13 ^c^	1.31 ± 0.15 ^d^	5.72 ± 0.38 ^b^	4.46	45.93	5.12	6.62
Ca	%	0.59 ± 0.04 ^c^	0.29 ± 0.01 ^c^	17.67 ± 1.54 ^a^	13.12 ± 0.49 ^b^	7.92	96.60	60.91	1.54
Fe	%	3.59 ± 0.24 ^ab^	2.04 ± 0.17 ^c^	4.18 ± 0.28 ^a^	3.40 ± 0.24 ^b^	3.30	23.78	2.05	2.94
K	%	2.41 ± 0.11 ^a^	0.79 ± 0.06 ^c^	0.25 ± 0.02 ^d^	1.15 ± 0.15 ^b^	1.15	69.09	9.63	1.86
Mg	%	0.62 ± 0.01 ^b^	0.27 ± 0.04 ^c^	3.39 ± 0.10 ^a^	0.73 ± 0.06 ^b^	1.25	99.48	12.55	0.78
Na	%	0.35 ± 0.03 ^a^	0.11 ± 0.01 ^bc^	0.14 ± 0.02 ^b^	0.08 ± 0.01 ^c^	0.17	61.19	4.26	1.02
Se	mg/kg	0.10 ± 0.00 ^b^	0.04 ± 0.00 ^b^	3.11 ± 0.16 ^a^	0.30 ± 0.02 ^b^	0.89	144.79	74.71	0.29
As	mg/kg	15.44 ± 0.33 ^b^	10.26 ± 0.38 ^b^	289.72 ± 6.53 ^a^	16.25 ± 0.38 ^b^	82.92	144.02	28.23	11.2
Mn	mg/kg	801.80 ± 9.42 ^a^	351.50 ± 7.19 ^c^	456.30 ± 11.18 ^b^	821.80 ± 10.30 ^a^	607.85	34.12	2.34	583
Ba	mg/kg	307.20 ± 6.71 ^a^	205.10 ± 3.96 ^b^	125.40 ± 7.09 ^c^	200.70 ± 5.40 ^b^	209.60	30.84	2.45	469
Cr	mg/kg	92.38 ± 1.10 ^a^	57.78 ± 1.03 ^c^	71.43 ± 4.33 ^b^	49.77 ± 6.19 ^c^	67.84	23.80	1.86	61
Cu	mg/kg	34.66 ± 2.20 ^b^	26.48 ± 1.48 ^b^	300.20 ± 6.12 ^a^	31.28 ± 3.23 ^b^	98.16	118.88	11.34	22.6
Li	mg/kg	57.52 ± 1.67 ^a^	36.70 ± 2.31 ^b^	15.90 ± 1.53 ^c^	41.49 ± 4.77 ^b^	37.90	39.21	3.62	32.5
Ni	mg/kg	69.23 ± 2.54 ^a^	21.23 ± 0.99 ^c^	19.78 ± 1.10 ^c^	32.61 ± 5.80 ^b^	35.71	55.94	3.50	26.9
Sr	mg/kg	42.43 ± 1.48 ^c^	33.15 ± 4.50 ^c^	124.30 ± 3.56 ^b^	179.10 ± 13.13 ^a^	94.75	63.59	5.40	167
V	mg/kg	134.50 ± 3.13 ^a^	82.50 ± 1.58 ^c^	90.88 ± 2.37 ^bc^	106.90 ± 9.00 ^b^	103.70	19.12	1.63	82.4
Zn	mg/kg	91.32 ± 2.28 ^b^	52.94 ± 2.07 ^c^	1120.00 ± 13.01 ^a^	89.48 ± 5.23 ^b^	338.44	133.41	21.16	74.2
Co	mg/kg	22.03 ± 1.52 ^a^	6.99 ± 0.30 ^b^	11.40 ± 1.94 ^b^	20.28 ± 2.10 ^a^	15.18	40.93	3.15	12.7
Ga	mg/kg	35.22 ± 1.68 ^a^	23.34 ± 0.57 ^b^	7.02 ± 1.01 ^c^	24.46 ± 2.50 ^b^	22.51	44.76	5.02	17.5
Mo	mg/kg	1.46 ± 0.05 ^b^	0.99 ± 0.02 ^b^	2.74 ± 0.33 ^a^	1.08 ± 0.13 ^b^	1.57	44.47	2.76	2
Cd	mg/kg	0.30 ± 0.03 ^b^	0.16 ± 0.01 ^b^	11.80 ± 1.32 ^a^	0.59 ± 0.07 ^b^	3.21	154.33	71.71	0.097
Cs	mg/kg	10.16 ± 0.56 ^b^	6.43 ± 0.24 ^c^	3.14 ± 0.21 ^d^	14.27 ± 1.58 ^a^	8.50	48.88	4.55	8.24
Pb	mg/kg	22.45 ± 0.70 ^b^	16.33 ± 0.23 ^b^	435.94 ± 5.49 ^a^	22.24 ± 2.61 ^b^	124.24	144.86	26.69	26
U	mg/kg	4.68 ± 0.19 ^a^	2.47 ± 0.23 ^b^	2.30 ± 0.18 ^b^	2.58 ± 0.42 ^b^	3.01	32.29	2.03	3.03

Note: Different superscript letters are used to indicate significant differences among villages (*p* < 0.05). “CV” Coefficient of variation; “Max” maximum; “Min” minimum.

**Table 4:** Statistical results of soil element contents in non-disease villages of SUD areas in Yunnan Province.

Elements	Units	Lishigeng	Songping	Gexi	Zejiu	Mean	CV (%)	Max/Min	Background Value [24]
Al	%	7.99 ± 0.16 ^a^	7.56 ± 0.23 ^a^	4.25 ± 0.14 ^c^	5.70 ± 0.33 ^b^	6.37	23.46	1.88	6.62
Ca	%	0.28 ± 0.02 ^b^	0.82 ± 0.08 ^b^	16.64 ± 1.11 ^a^	0.39 ± 0.04 ^b^	4.53	154.38	60.33	1.54
Fe	%	3.92 ± 0.15 ^a^	3.30 ± 0.19 ^b^	2.52 ± 0.17 ^c^	3.00 ± 0.10 ^bc^	3.18	15.99	1.56	2.94
K	%	2.37 ± 0.05 ^a^	2.09 ± 0.12 ^b^	0.57 ± 0.05 ^d^	0.97 ± 0.05 ^c^	1.50	50.09	4.19	1.86
Mg	%	0.78 ± 0.06 ^a^	0.93 ± 0.12 ^a^	0.31 ± 0.03 ^b^	0.38 ± 0.05 ^b^	0.60	43.22	2.96	0.78
Na	%	0.66 ± 0.06 ^b^	0.94 ± 0.06 ^a^	0.28 ± 0.03 ^c^	0.10 ± 0.01 ^d^	0.50	66.28	3.35	1.02
Se	mg/kg	0.06 ± 0.01 ^b^	0.04 ± 0.01 ^b^	1.41 ± 0.13 ^a^	0.08 ± 0.01 ^b^	0.40	147.20	33.60	0.29
As	mg/kg	30.16 ± 1.13 ^a^	13.37 ± 0.61 ^b^	16.50 ± 0.65 ^b^	32.92 ± 2.31 ^a^	23.24	36.30	2.46	11.2
Mn	mg/kg	958.30 ± 11.30 ^a^	551.40 ± 7.89 ^b^	188.60 ± 3.31 ^c^	210.10 ± 6.22 ^c^	477.10	65.58	5.08	583
Ba	mg/kg	297.80 ± 8.66 ^b^	652.00 ± 10.17 ^a^	50.14 ± 1.54 ^d^	168.50 ± 5.69 ^c^	292.11	77.19	13.00	469
Cr	mg/kg	59.16 ± 4.23 ^a^	52.43 ± 1.88 ^ab^	56.01 ± 1.71 ^a^	43.54 ± 2.83 ^b^	52.79	11.07	1.36	61
Cu	mg/kg	43.27 ± 2.19 ^b^	53.20 ± 2.65 ^a^	0.51 ± 0.10 ^d^	28.51 ± 1.66 c	31.37	63.32	104.13	22.6
Li	mg/kg	58.32 ± 2.59 ^a^	56.25 ± 1.73 ^a^	52.15 ± 2.70 ^a^	35.68 ± 4.28 ^b^	50.60	17.58	1.63	32.5
Ni	mg/kg	56.50 ± 1.50 ^a^	34.16 ± 2.09 ^b^	25.18 ± 1.53 ^c^	29.49 ± 2.18 ^bc^	36.33	33.22	2.24	26.9
Sr	mg/kg	61.30 ± 2.05 ^c^	107.20 ± 1.73 ^b^	904.90 ± 12.68 ^a^	33.62 ± 3.14 ^d^	276.76	131.38	26.92	167
V	mg/kg	140.70 ± 2.75 ^a^	109.60 ± 2.86 ^c^	127.00 ± 4.62 ^b^	109.70 ± 2.99 ^c^	121.75	10.71	1.28	82.4
Zn	mg/kg	89.51 ± 4.16 ^b^	262.50 ± 8.11 ^a^	38.31 ± 4.33 ^d^	59.09 ± 4.14 ^c^	112.35	78.84	6.85	74.2
Co	mg/kg	30.62 ± 1.82 ^a^	18.15 ± 0.94 ^b^	8.96 ± 0.65 ^c^	11.99 ± 1.94 ^c^	17.43	47.64	3.42	12.7
Ga	mg/kg	41.79 ± 2.83 ^a^	42.27 ± 1.76 ^a^	16.09 ± 1.19 ^c^	23.52 ± 2.16 ^b^	30.92	36.94	2.63	17.5
Mo	mg/kg	3.45 ± 0.14 ^b^	1.55 ± 0.12 ^c^	1.68 ± 0.16 ^c^	4.87 ± 0.42 ^a^	2.89	47.40	3.15	2
Cd	mg/kg	0.26 ± 0.03 ^ab^	0.43 ± 0.08 ^a^	0.24 ± 0.09 ^ab^	0.10 ± 0.01 ^b^	0.26	45.68	4.36	0.097
Cs	mg/kg	12.40 ± 1.05 ^b^	11.38 ± 1.02 ^b^	5.75 ± 0.22 ^c^	17.11 ± 1.53 ^a^	11.66	34.62	2.97	8.24
Pb	mg/kg	31.37 ± 1.94 ^b^	36.97 ± 1.04 ^a^	15.42 ± 0.62 ^c^	17.33 ± 0.95 ^c^	25.28	36.16	2.40	26
U	mg/kg	4.28 ± 0.17 ^a^	3.09 ± 0.04 ^b^	2.33 ± 0.16 ^c^	3.62 ± 0.37 ^ab^	3.33	21.52	1.84	3.03

Note: Different superscript letters are used to indicate significant differences among villages (*p* < 0.05). “CV” Coefficient of variation; “Max” maximum; “Min” minimum.

**Table 5:** Statistics on the content of elements in corn in the disease villages of the SUD areas in Yunnan Province.

Elements	Units	A’jiju	Cangdi	Huakou	Xiaolongtan	Mean	CV (%)	Max/Min	GB2762-2017 [28]	NY861-2004 [27]
Ca	mg/kg	61.00 ± 5.83 ^b^	70.50 ± 1.30 ^b^	118.80 ± 3.16 ^a^	70.75 ± 2.64 ^b^	80.26	28.15	1.95	ND	ND
K	mg/kg	2889.00 ± 52.89 ^c^	2667.00 ± 11.06 ^d^	3462.00 ± 26.15 ^b^	4084.00 ± 32.73 ^a^	3275.50	16.78	1.53	ND	ND
Mg	mg/kg	1221.00 ± 10.46 ^b^	1376.00 ± 13.11 ^a^	1130.00 ± 33.32 ^c^	1217.00 ± 16.17 ^b^	1236.00	7.17	1.22	ND	ND
Na	mg/kg	48.19 ± 2.01 ^a^	22.74 ± 0.93 ^c^	31.36 ± 2.44 ^b^	28.25 ± 1.95 ^bc^	32.64	29.10	2.12	ND	ND
P	mg/kg	2573.00 ± 12.59 ^c^	3285.00 ± 20.21 ^a^	2239.00 ± 25.53 ^d^	2902.00 ± 12.41 ^b^	2749.75	14.11	1.47	ND	ND
Al	mg/kg	31.20 ± 2.43 ^a^	22.79 ± 1.39 ^bc^	17.49 ± 1.11 ^c^	23.49 ± 1.30 ^b^	23.74	20.60	1.78	ND	ND
Ba	mg/kg	0.13 ± 0.02 ^a^	0.13 ± 0.02 ^a^	0.14 ± 0.02 ^a^	0.15 ± 0.02 ^a^	0.14	5.41	1.14	ND	ND
Cr	mg/kg	0.64 ± 0.03 ^ab^	0.51 ± 0.08 ^b^	0.88 ± 0.10 ^a^	0.59 ± 0.08 ^b^	0.66	21.25	1.74	1	1
Cu	mg/kg	0.98 ± 0.04 ^b^	2.01 ± 0.29 ^a^	1.73 ± 0.15 ^a^	1.95 ± 0.04 ^a^	1.67	24.75	2.06	ND	10
Fe	mg/kg	12.34 ± 0.34 ^b^	17.54 ± 1.28 ^a^	15.93 ± 1.11 ^ab^	19.52 ± 2.29 ^a^	16.33	16.12	1.58	ND	ND
Mn	mg/kg	3.92 ± 0.06 ^b^	5.51 ± 0.28 ^a^	3.77 ± 0.35 ^b^	3.79 ± 0.15 ^b^	4.25	17.26	1.46	ND	ND
Sr	mg/kg	0.14 ± 0.02 ^a^	0.15 ± 0.02 ^a^	0.21 ± 0.07 ^a^	0.12 ± 0.02 ^a^	0.16	21.27	1.70	ND	ND
Zn	mg/kg	12.79 ± 2.70 ^b^	17.21 ± 1.15 ^ab^	18.78 ± 1.12 ^a^	21.93 ± 1.87 ^a^	17.68	18.63	1.71	ND	50
Co	mg/kg	0.01 ± 0.00 ^a^	0.00 ± 0.00 ^b^	0.01 ± 0.00 ^a^	0.00 ± 0.00 ^b^	0.01	31.85	2.35	ND	ND
Se	μg/kg	4.67 ± 0.27 ^b^	5.47 ± 0.27 ^b^	16.18 ± 1.36 ^a^	4.28 ± 0.05 ^b^	7.65	64.56	3.78	ND	300
As	μg/kg	0.05 ± 0.01 ^b^	0.00 ± 0.00 ^b^	3.54 ± 0.26 ^a^	0.00 ± 0.00 ^b^	0.90	170.01	ND	500	700
Li	μg/kg	10.26 ± 0.49 ^b^	4.02 ± 0.17 ^c^	11.61 ± 0.56 ^b^	20.99 ± 1.49 ^a^	11.72	51.79	5.22	ND	ND
V	μg/kg	3421.11 ± 85.19 ^a^	2645.46 ± 12.57 ^b^	2678.23 ± 69.21 ^b^	1494.75 ± 40.69 ^c^	2559.89	26.91	2.29	ND	ND
Ni	μg/kg	51.48 ± 2.27 ^c^	46.06 ± 2.04 ^c^	137.70 ± 6.16 ^b^	416.13 ± 9.82 ^a^	162.84	92.53	9.04	1000	ND
Ga	μg/kg	9.73 ± 0.37 ^a^	9.90 ± 0.36 ^a^	8.01 ± 0.52 ^b^	8.26 ± 0.16 ^b^	8.97	9.45	1.24	ND	ND
Rb	μg/kg	456.47 ± 8.03 ^c^	570.89 ± 5.78 ^c^	3395.71 ± 75.79 ^b^	14,202.98 ± 75.22 ^a^	4656.51	121.03	31.11	ND	ND
Mo	μg/kg	668.70 ± 10.27 ^b^	424.35 ± 7.83 ^c^	708.08 ± 10.53 ^a^	406.46 ± 9.33 ^c^	551.90	24.89	1.74	ND	ND
Cd	μg/kg	2.69 ± 0.16 ^c^	2.75 ± 0.50 ^c^	7.73 ± 0.45 ^a^	4.00 ± 0.13 ^b^	4.29	47.76	2.87	100	50
Cs	μg/kg	0.57 ± 0.04 ^c^	0.63 ± 0.08 ^c^	28.31 ± 2.18 ^b^	112.30 ± 4.35 ^a^	35.45	129.15	197.19	ND	ND
Pb	μg/kg	43.13 ± 2.02 ^b^	18.10 ± 0.91 ^c^	75.11 ± 5.06 ^a^	38.78 ± 1.24 ^b^	43.78	46.63	4.15	200	400
U	μg/kg	0.78 ± 0.04 ^a^	0.42 ± 0.07 ^b^	0.83 ± 0.09 ^a^	0.50 ± 0.03 ^b^	0.63	27.47	1.96	ND	ND

Note: “ND” no data. Different superscript letters are used to indicate significant differences among villages (*p* < 0.05). “CV” Coefficient of variation; “Max” maximum; “Min” minimum.

**Table 6:** Statistical results of corn element content in non-disease villages of the SUD areas in Yunnan Province.

Elements	Units	Lishigeng	Songping	Gexi	Zejiu	Mean	CV (%)	Max/Min	GB2762-2017 [28]	NY861-2004 [27]
Ca	mg/kg	71.34 ± 3.56 ^b^	57.98 ± 2.06 ^c^	68.48 ± 2.12 ^bc^	110.20 ± 5.90 ^a^	77.00	25.72	1.90	ND	ND
K	mg/kg	3971.00 ± 92.66 ^a^	1552.00 ± 27.65 ^d^	1978.00 ± 37.44 ^c^	2471.00 ± 33.50 ^b^	2493.00	36.63	2.56	ND	ND
Mg	mg/kg	1296.00 ± 23.46 ^a^	965.60 ± 44.04 ^c^	877.90 ± 21.85 ^c^	1067.00 ± 23.46 ^b^	1051.63	14.85	1.22	ND	ND
Na	mg/kg	34.61 ± 1.30 ^ab^	26.41 ± 2.92 ^b^	30.75 ± 3.26 ^ab^	36.46 ± 3.22 ^a^	32.06	12.03	1.38	ND	ND
P	mg/kg	3158.00 ± 24.76 ^a^	2632.00 ± 33.50 ^b^	2166.00 ± 30.18 ^c^	2395.00 ± 130.79 ^c^	2587.75	14.23	1.46	ND	ND
Al	mg/kg	7.97 ± 0.48 ^c^	14.81 ± 1.08 ^b^	1.89 ± 0.13 ^d^	38.18 ± 3.07 ^a^	15.71	87.54	20.22	ND	ND
Ba	mg/kg	0.38 ± 0.04 ^a^	0.21 ± 0.08 ^b^	0.05 ± 0.01 ^c^	0.17 ± 0.01 ^bc^	0.20	57.45	7.41	ND	ND
Cr	mg/kg	0.62 ± 0.08 ^a^	0.61 ± 0.14 ^a^	0.44 ± 0.06 ^a^	0.58 ± 0.05 ^a^	0.56	12.85	1.41	1	1
Cu	mg/kg	1.34 ± 0.09 ^c^	1.83 ± 0.12 ^bc^	2.25 ± 0.13 ^ab^	2.63 ± 0.29 ^a^	2.01	23.97	1.97	ND	10
Fe	mg/kg	11.76 ± 0.54 ^b^	12.87 ± 1.06 ^b^	17.00 ± 1.02 ^a^	19.10 ± 0.42 ^a^	15.18	19.68	1.62	ND	ND
Mn	mg/kg	5.12 ± 0.17 ^a^	4.53 ± 0.46 ^a^	3.25 ± 0.31 ^b^	2.92 ± 0.29 ^b^	3.95	22.83	1.75	ND	ND
Sr	mg/kg	0.14 ± 0.02 ^b^	0.13 ± 0.02 ^b^	0.09 ± 0.01 ^b^	0.27 ± 0.03 ^a^	0.16	43.80	3.09	ND	ND
Zn	mg/kg	14.59 ± 0.43 ^c^	21.93 ± 1.21 ^a^	17.03 ± 0.68 ^bc^	17.79 ± 0.73 ^b^	17.84	14.82	1.50	ND	50
Co	mg/kg	0.02 ± 0.00 ^c^	0.07 ± 0.00 ^a^	0.04 ± 0.01 ^b^	0.02 ± 0.00 ^c^	0.04	51.82	3.31	ND	ND
Se	μg/kg	0.00 ± 0.00 ^c^	2.16 ± 0.15 ^b^	17.64 ± 1.03 ^a^	1.44 ± 0.12 ^bc^	5.31	134.87	ND	ND	300
As	μg/kg	0.00 ± 0.00 ^d^	3.01 ± 0.17 ^c^	5.35 ± 0.35 ^b^	9.55 ± 0.35 ^a^	4.48	77.92	ND	500	700
Li	μg/kg	5.92 ± 0.26 ^c^	7.03 ± 1.66 ^c^	10.13 ± 0.63 ^b^	13.31 ± 22.86 ^a^	9.10	31.69	2.25	ND	ND
V	μg/kg	2828.78 ± 22.86 ^b^	1935.19 ± 60.98 ^c^	2817.45 ± 109.55 ^b^	3368.29 ± 33.09 ^a^	2737.43	18.77	1.74	ND	ND
Ni	μg/kg	322.89 ± 16.97 ^a^	273.98 ± 12.88 ^b^	200.17 ± 10.86 ^c^	201.89 ± 13.25 ^c^	249.73	20.70	1.61	1000	ND
Ga	μg/kg	9.29 ± 0.52 ^b^	7.51 ± 0.41 ^bc^	6.90 ± 0.45 ^c^	11.93 ± 1.08 ^a^	8.91	21.92	1.73	ND	ND
Rb	μg/kg	764.28 ± 16.48 ^c^	658.36 ± 19.45 ^c^	2112.16 ± 47.88 ^a^	1940.62 ± 39.45 ^b^	1368.86	48.32	3.21	ND	ND
Mo	μg/kg	289.90 ± 12.88 ^c^	181.34 ± 10.85 ^d^	679.38 ± 23.30 ^b^	764.55 ± 21.48 ^a^	478.79	51.80	4.22	ND	ND
Cd	μg/kg	1.67 ± 0.12 ^c^	2.09 ± 0.40 ^c^	19.20 ± 1.05 ^a^	12.59 ± 1.27 ^b^	8.89	83.13	11.52	100	50
Cs	μg/kg	1.45 ± 0.07 ^b^	1.36 ± 0.18 ^b^	16.13 ± 1.55 ^a^	17.66 ± 0.67 ^a^	9.15	84.82	12.97	ND	ND
Pb	μg/kg	24.85 ± 1.31 ^c^	25.68 ± 2.92 ^c^	35.78 ± 1.78 ^b^	99.85 ± 3.77 ^a^	46.54	66.77	4.02	200	400
U	μg/kg	0.44 ± 0.09 ^b^	0.41 ± 0.07 ^b^	0.43 ± 0.07 ^b^	1.52 ± 0.10 ^a^	0.70	67.67	3.69	ND	ND

Note: “ND” no data. Different superscript letters are used to indicate significant differences among villages (*p* < 0.05). “CV” Coefficient of variation; “Max” maximum; “Min” minimum.

**Table 7:** Statistical results of element content in drinking water in disease villages of the SUD area in Yunnan Province.

Elements	Units	A’jiju	Cangdi	Mean	CV (%)	Max/Min	GB5749-2022 [32]	WHO [33]
Ca	mg/L	153.80 ± 4.31 ^a^	50.44 ± 2.68 ^b^	102.12	50.61	3.05	ND	ND
K	mg/L	1.75 ± 0.12 ^a^	0.74 ± 0.08 ^b^	1.24	40.56	2.36	ND	ND
Mg	mg/L	23.72 ± 1.68 ^a^	9.48 ± 0.32 ^b^	16.60	42.90	2.50	ND	ND
Na	mg/L	9.70 ± 0.76 ^a^	5.23 ± 0.17 ^b^	7.46	29.91	1.85	200	200
P	μg/L	70.20 ± 1.53 ^a^	77.80 ± 2.36 ^a^	74.00	5.14	1.11	ND	ND
Al	μg/L	0.80 ± 0.09 ^a^	0.00 ± 0.00 ^b^	0.40	100.00	ND	200	200
Fe	μg/L	3.40 ± 0.19 ^b^	9.20 ± 0.51 ^a^	6.30	46.03	2.71	300	300
Sr	μg/L	2129.00 ± 31.39 ^a^	389.80 ± 11.30 ^b^	1259.40	69.05	5.46	ND	ND
Li	μg/L	4.91 ± 0.25 ^a^	3.23 ± 0.14 ^b^	4.07	20.72	1.52	ND	ND
V	μg/L	0.61 ± 0.09 ^b^	2.16 ± 0.32 ^a^	1.39	55.94	3.54	ND	ND
Cr	μg/L	0.78 ± 0.08 ^a^	0.69 ± 0.10 ^a^	0.73	5.82	1.12	50	50
Mn	μg/L	1.60 ± 0.09 ^a^	1.16 ± 0.09 ^b^	1.38	16.03	1.38	100	400
Co	μg/L	0.05 ± 0.01 ^a^	0.04 ± 0.01 ^a^	0.04	9.81	1.22	ND	ND
Ni	μg/L	1.15 ± 0.10 ^a^	0.55 ± 0.05 ^b^	0.85	35.51	2.10	20	70
Cu	μg/L	0.78 ± 0.11 ^a^	0.45 ± 0.04 ^b^	0.61	26.13	1.71	1000	2000
Zn	μg/L	12.57 ± 0.88 ^b^	325.61 ± 4.94 ^a^	169.09	92.57	25.90	1000	3000
Ga	μg/L	0.01 ± 0.00 ^a^	0.01 ± 0.00 ^a^	0.01	9.32	1.21	ND	ND
As	μg/L	0.58 ± 0.05 ^b^	0.84 ± 0.07 ^a^	0.71	17.95	1.44	10	10
Se	μg/L	0.00 ± 0.00 ^b^	0.85 ± 0.07 ^a^	0.42	100.00	ND	10	40
Rb	μg/L	0.68 ± 0.06 ^a^	0.39 ± 0.04 ^b^	0.53	26.83	1.73	ND	ND
Mo	μg/L	2.71 ± 0.09 ^a^	0.11 ± 0.02 ^b^	1.41	92.44	25.46	70	70
Cd	μg/L	0.01 ± 0.00 ^a^	0.01 ± 0.00 ^a^	0.01	11.79	1.27	5	3
Cs	μg/L	0.01 ± 0.00 ^a^	0.00 ± 0.00 ^b^	0.00	53.49	3.30	ND	ND
Ba	μg/L	80.86 ± 0.00 ^b^	166.30 ± 0.00 ^a^	123.58	34.57	2.06	700	700
Pb	μg/L	0.04 ± 0.00 ^a^	0.04 ± 0.00 ^a^	0.04	4.80	1.10	10	10
U	μg/L	1.25 ± 0.10 ^a^	0.35 ± 0.05 ^b^	0.80	56.72	3.62	ND	30

Note: “ND” no data. Different superscript letters are used to indicate significant differences among villages (*p* < 0.05). “CV” Coefficient of variation; “Max” maximum; “Min” minimum.

**Table 8:** Statistical results of element content in drinking water in non-disease villages of the SUD area in Yunnan Province.

Elements	Units	Lishigeng	Songping	Gexi	Zejiu	Mean	CV (%)	Max/Min	GB5749-2022 [32]	WHO [33]
Ca	mg/L	69.69 ± 1.76 ^b^	6.63 ± 0.07 ^c^	91.76 ± 2.51 ^a^	91.85 ± 1.33 ^a^	64.98	53.68	13.86	ND	ND
K	mg/L	0.89 ± 0.05 ^b^	1.30 ± 0.13 ^a^	0.94 ± 0.08 ^b^	1.16 ± 0.08 ^ab^	1.07	15.65	1.47	ND	ND
Mg	mg/L	11.70 ± 0.81 ^b^	2.02 ± 0.16 ^c^	14.25 ± 0.89 ^a^	14.73 ± 0.74 ^a^	10.68	48.02	7.28	ND	ND
Na	mg/L	8.46 ± 0.23 ^a^	2.39 ± 0.13 ^b^	1.80 ± 0.11 ^c^	1.65 ± 0.12 ^c^	3.57	79.26	5.13	200	200
P	μg/L	60.70 ± 1.00 ^b^	119.30 ± 9.53 ^a^	27.90 ± 1.03 ^c^	31.30 ± 1.65 ^c^	59.80	61.28	4.28	ND	ND
Al	μg/L	0.00 ± 0.00 ^b^	2.40 ± 0.19 ^b^	0.10 ± 0.01 ^b^	33.70 ± 1.95 ^a^	9.05	157.61	ND	200	200
Fe	μg/L	7.90 ± 0.13 ^b^	11.10 ± 1.06 ^a^	0.50 ± 0.04 ^c^	2.20 ± 0.28 ^c^	5.43	78.74	22.20	300	300
Sr	μg/L	735.00 ± 14.99 ^a^	42.80 ± 2.64 ^c^	51.00 ± 1.47 ^c^	208.10 ± 5.24 ^b^	259.23	108.97	17.17	ND	ND
Li	μg/L	5.85 ± 0.11 ^a^	4.25 ± 0.26 ^b^	0.20 ± 0.06 ^c^	0.67 ± 0.07 ^c^	2.74	86.88	29.83	ND	ND
V	μg/L	0.37 ± 0.03 ^c^	0.79 ± 0.06 ^b^	1.19 ± 0.09 ^a^	0.92 ± 0.11 ^b^	0.82	36.27	3.23	ND	ND
Cr	μg/L	0.62 ± 0.05 ^c^	0.74 ± 0.08 ^bc^	1.59 ± 0.06 ^a^	0.96 ± 0.13 ^b^	0.98	38.07	2.54	50	50
Mn	μg/L	1.90 ± 0.13 ^b^	3.27 ± 0.21 ^a^	0.13 ± 0.01 ^c^	0.40 ± 0.04 ^c^	1.42	88.68	25.43	100	400
Co	μg/L	0.06 ± 0.01 ^a^	0.02 ± 0.00 ^c^	0.03 ± 0.00 ^bc^	0.04 ± 0.01 ^ab^	0.04	42.17	3.66	ND	ND
Ni	μg/L	0.57 ± 0.06 ^c^	0.40 ± 0.07 ^c^	2.20 ± 0.14 ^a^	1.27 ± 0.05 ^b^	1.11	64.12	5.56	20	70
Cu	μg/L	1.72 ± 0.08 ^a^	1.41 ± 0.14 ^b^	0.17 ± 0.03 ^c^	1.83 ± 0.10 ^a^	1.28	51.76	11.08	1000	2000
Zn	μg/L	2809.21 ± 10.63 ^a^	71.85 ± 1.85 ^b^	8.79 ± 0.10 ^c^	25.49 ± 0.82 ^c^	728.83	164.83	319.67	1000	3000
Ga	μg/L	0.01 ± 0.00 ^b^	0.00 ± 0.00 ^c^	0.00 ± 0.00 ^b^	0.01 ± 0.00 ^a^	0.01	69.88	13.56	ND	ND
As	μg/L	0.49 ± 0.02 ^a^	0.41 ± 0.05 ^ab^	0.14 ± 0.01 ^c^	0.33 ± 0.03 ^b^	0.35	37.30	3.42	10	10
Se	μg/L	0.00 ± 0.00 ^c^	0.00 ± 0.00 ^c^	1.56 ± 0.05 ^a^	0.13 ± 0.01 ^b^	0.42	155.46	ND	10	40
Rb	μg/L	0.52 ± 0.07 ^c^	1.42 ± 0.11 ^a^	1.32 ± 0.10 ^a^	0.89 ± 0.06 ^b^	1.04	34.57	2.73	ND	ND
Mo	μg/L	0.83 ± 0.08 ^b^	0.01 ± 0.00 ^c^	0.21 ± 0.01 ^c^	1.24 ± 0.12 ^a^	0.57	85.78	172.56	70	70
Cd	μg/L	0.01 ± 0.00 ^c^	0.00 ± 0.00 ^d^	0.07 ± 0.00 ^a^	0.03 ± 0.00 ^b^	0.03	84.93	15.07	5	3
Cs	μg/L	0.02 ± 0.00 ^a^	0.00 ± 0.00 ^c^	0.02 ± 0.00 ^b^	0.02 ± 0.00 ^a^	0.02	42.00	4.33	ND	ND
Ba	μg/L	82.49 ± 1.75 ^a^	31.27 ± 1.32 ^b^	8.35 ± 0.18 ^d^	16.11 ± 0.19 ^c^	34.55	83.57	9.87	700	700
Pb	μg/L	0.09 ± 0.01 ^b^	0.28 ± 0.02 ^a^	0.01 ± 0.00 ^c^	0.09 ± 0.01 ^b^	0.12	85.33	30.47	10	10
U	μg/L	0.32 ± 0.02 ^c^	0.00 ± 0.00 ^d^	0.87 ± 0.04 ^a^	0.44 ± 0.03 ^b^	0.41	76.06	198.52	ND	30

Note: “ND” no data. Different superscript letters are used to indicate significant differences among villages (*p* < 0.05). “CV” Coefficient of variation; “Max” maximum; “Min” minimum.

## Data Availability

The raw data supporting the conclusions of this article will be made available by the authors on request.

## Supplementary Materials

The following supporting information can be downloaded at: https://www.mdpi.com/article/10.3390/toxics13121078/s1, Table S1: The operating parameter for ICP-OES instrument; Table S2: The operating parameter for ICP-MS instrument; Table S3: The operating parameter for ICP-MS/MS instrument; Table S4: The operating parameter for HG-AFS instrument; Table S5: The *t*-test results of the average concentrations of soil elements in typical disease villages and adjacent non-disease villages in the area with SUD in Yunnan Province; Table S6: The *t*-test results of the average concentrations of corn elements in typical disease villages and adjacent non-disease villages in the area with SUD in Yunnan Province; Table S7: The results of the *t*-test for the average element concentrations of drinking water in typical disease villages and adjacent non-disease villages in SUD area of Yunnan Province; Table S8: The *T*-test results of the average element concentrations of soil in the KD villages in Shaanxi Province and SUD villages in Yunnan Province; Table S9: The *t*-test results of the average element concentrations of corn in the KD villages in Shaanxi Province and SUD villages in Yunnan Province; Table S10: The *t*-test results of the average element concentrations of drinking water in the KD villages in Shaanxi Province and SUD villages in Yunnan Province; Table S11: The statistical results of the element hazard quotient (HQ) in corn for residents of the disease villages in SUD area of Yunnan Province; Table S12: The statistical results of the element hazard quotient (HQ) in drinking water for residents of the disease villages in SUD area of Yunnan Province; Table S13: The statistical results of the element hazard quotient (HQ) in corn for residents of the non-disease villages in SUD area of Yunnan Province; Table S14: The statistical results of the element hazard quotient (HQ) in drinking water for residents of the non-disease villages in SUD area of Yunnan Province.
