# Supplementary material for: Toxicological Assessment of Trace Element Exposure in Relation to Sudden Unexplained Death (SUD): Environmental Geochemistry and Dietary Risk in Central-Eastern Yunnan, China
Source: Toxics. 2025 Dec 14;13(12):1078. doi: 10.3390/toxics13121078 (PMC12737347; doi:10.3390/toxics13121078)
Supplement: Supplementary file 1 [file toxics-13-01078-s001.zip › toxics-4010088-supplementary.pdf]

Table S1. The operating parameter for ICP-OES instrument

Parameter	Value
RF power (W)	1300
Cool gas flow (L/min)	12
Auxiliary gas flow (L/min)	0.2
Nebulizer gas flow (L/min)	0.55
Sample uptake rate (mL/min)	1

Table S2. The operating parameter for ICP-MS instrument

Parameter	Value
RF power (W)	1300
Cool gas flow (L/min)	12
Auxiliary gas flow (L/min)	1.8
Nebulizer gas flow (L/min)	0.8
Sample uptake rate (mL/min)	0.5
Torch	Shield torch
Cones	Nickel
Dwell time (ms)	10
Resolution	Standard
Analogue detector voltage (V)	3750
PC detector voltage (V)	1950

Table S3. The operating parameter for ICP-MS/MS instrument.

Parameter	Value
Scan mode	MS/MS
RF power (W)	1550
Sampling depth (mm)	9
Nebulizer gas flow (L/min)	0.7
Make-up gas flow (L/min)	0.4
Extract 1 (V)	0
Extract 1 (V)	-180
Cell gas flow (mL/min)	O_2=0.4
Q1/Q2	78/94 (Se)

Table S4. The operating parameter for HG-AFS instrument.

Parameter	Value
Negative high voltage (V)	280
Lamp current (mA)	80
Atomizer height (mm)	8
Atomizer temperature (°C)	200
Carrier gas flow (mL/min)	400
Shielded gas flow (mL/min)	800
Reading duration (s)	7.0
Delay time (s)	1.5

Table S5. The T-test results of the average concentrations of soil elements in typical disease villages and adjacent non-disease villages in the area with SUD in Yunnan Province

Elements	Units	Processing 1	Processing 2	Mean (Processing 1)	Mean (Processing 2)	P value	Significance
Al	%	Disease village	Non-disease village	4.46	6.37	0.02	*
Ca	%	Disease village	Non-disease village	7.92	4.53	0.26	ns
Fe	%	Disease village	Non-disease village	3.30	3.18	0.71	ns
K	%	Disease village	Non-disease village	1.15	1.50	0.26	ns
Mg	%	Disease village	Non-disease village	1.25	0.60	0.14	ns
Na	%	Disease village	Non-disease village	0.17	0.50	0.01	**
Se	mg/kg	Disease village	Non-disease village	0.89	0.40	0.35	ns
As	mg/kg	Disease village	Non-disease village	82.92	23.24	0.45	ns
Mn	mg/kg	Disease village	Non-disease village	607.85	477.10	0.10	ns
Ba	mg/kg	Disease village	Non-disease village	209.60	292.11	0.97	ns
Cr	mg/kg	Disease village	Non-disease village	67.84	52.79	0.02	*
Cu	mg/kg	Disease village	Non-disease village	98.16	31.37	0.05	*
Li	mg/kg	Disease village	Non-disease village	37.90	50.60	0.03	*
Ni	mg/kg	Disease village	Non-disease village	35.71	36.33	0.56	ns
Sr	mg/kg	Disease village	Non-disease village	94.75	276.76	0.29	ns
V	mg/kg	Disease village	Non-disease village	103.70	121.75	0.03	*
Zn	mg/kg	Disease village	Non-disease village	338.44	112.35	0.20	ns
Co	mg/kg	Disease village	Non-disease village	15.18	17.43	0.50	ns
Ga	mg/kg	Disease village	Non-disease village	22.51	30.92	0.09	ns
Mo	mg/kg	Disease village	Non-disease village	1.57	2.89	0.01	**
Cd	mg/kg	Disease village	Non-disease village	3.21	0.26	0.05	*
Cs	mg/kg	Disease village	Non-disease village	8.50	11.66	0.10	ns
Pb	mg/kg	Disease village	Non-disease village	124.24	25.28	0.16	ns

U	mg/kg	Disease village	Non-disease village	3.01	3.33	0.33	ns

Note: "ns" non-significant; **$P < 0.01$ and *$P < 0.05$ indicate that there are significant differences in the average element concentrations between the disease villages and non-disease villages

Table S6. The T-test results of the average concentrations of corn elements in typical disease villages and adjacent non-disease villages in the area with SUD in Yunnan Province

Elements	Units	Processing 1	Processing 2	Mean (Processing 1)	Mean (Processing 2)	P value	Significance
Ca	mg/kg	Disease village	Non-disease village	80.26	77.00	0.75	ns
K	mg/kg	Disease village	Non-disease village	3275.50	2493.00	0.01	*
Mg	mg/kg	Disease village	Non-disease village	1236.00	1051.63	0.00	**
Na	mg/kg	Disease village	Non-disease village	32.64	32.06	0.87	ns
P	mg/kg	Disease village	Non-disease village	2749.75	2587.75	0.33	ns
Al	mg/kg	Disease village	Non-disease village	23.74	15.71	0.02	*
Ba	mg/kg	Disease village	Non-disease village	0.14	0.20	0.13	ns
Cr	mg/kg	Disease village	Non-disease village	0.66	0.56	0.20	ns
Cu	mg/kg	Disease village	Non-disease village	1.67	2.01	0.13	ns
Fe	mg/kg	Disease village	Non-disease village	16.33	15.18	0.42	ns
Mn	mg/kg	Disease village	Non-disease village	4.25	3.95	0.46	ns
Sr	mg/kg	Disease village	Non-disease village	0.16	0.16	0.94	ns
Zn	mg/kg	Disease village	Non-disease village	17.68	17.84	0.92	ns
Co	mg/kg	Disease village	Non-disease village	0.01	0.04	0.00	***
Se	μg/kg	Disease village	Non-disease village	7.65	5.31	0.04	*
As	μg/kg	Disease village	Non-disease village	0.90	4.48	0.01	**
Li	μg/kg	Disease village	Non-disease village	11.72	9.10	0.43	ns
V	μg/kg	Disease village	Non-disease village	2559.89	2737.43	0.50	ns
Ni	μg/kg	Disease village	Non-disease village	162.84	249.73	0.01	**
Ga	μg/kg	Disease village	Non-disease village	8.97	8.91	0.74	ns

Rb	μg/kg	Disease village	Non-disease village	4656.51	1368.86	0.33	ns
Mo	μg/kg	Disease village	Non-disease village	551.90	478.79	0.18	ns
Cd	μg/kg	Disease village	Non-disease village	4.29	8.89	0.27	ns
Cs	μg/kg	Disease village	Non-disease village	35.45	9.15	0.55	ns
Pb	μg/kg	Disease village	Non-disease village	43.78	46.54	0.99	ns
U	μg/kg	Disease village	Non-disease village	0.63	0.70	0.88	ns

Note: "ns" non-significant; ***$P < 0.001$,**$P < 0.01$ and *$P < 0.05$ indicate that there are significant differences in the average element concentrations between the disease villages and non-disease villages

Table S7. The results of the t-test for the average element concentrations of drinking water in typical disease villages and adjacent non-disease villages in SUD area of Yunnan Province

Elements	Units	Processing 1	Processing 2	Mean (Processing 1)	Mean (Processing 2)	P value	Significance
Ca	mg/L	Disease village	Non-disease village	102.12	38.16	0.04	*
K	mg/L	Disease village	Non-disease village	1.24	1.09	0.58	ns
Mg	mg/L	Disease village	Non-disease village	16.60	6.86	0.03	*
Na	mg/L	Disease village	Non-disease village	7.46	5.42	0.19	ns
P	μg/L	Disease village	Non-disease village	74.00	90.00	0.40	ns
Al	μg/L	Disease village	Non-disease village	0.40	1.20	0.32	ns
Fe	μg/L	Disease village	Non-disease village	6.30	9.50	0.07	ns
Sr	μg/L	Disease village	Non-disease village	1259.40	388.90	0.05	ns
Li	μg/L	Disease village	Non-disease village	4.07	5.05	0.10	ns
V	μg/L	Disease village	Non-disease village	1.39	0.58	0.06	ns
Cr	μg/L	Disease village	Non-disease village	0.73	0.68	0.52	ns
Mn	μg/L	Disease village	Non-disease village	1.38	2.59	0.00	**
Co	μg/L	Disease village	Non-disease village	0.04	0.04	0.48	ns
Ni	μg/L	Disease village	Non-disease village	0.85	0.48	0.04	*
Cu	μg/L	Disease village	Non-disease village	0.61	1.56	0.00	***

Zn	μg/L	Disease village	Non-disease village	169.09	1440.53	0.11	ns
Ga	μg/L	Disease village	Non-disease village	0.01	0.00	0.00	**
As	μg/L	Disease village	Non-disease village	0.71	0.45	0.01	**
Se	μg/L	Disease village	Non-disease village	0.42	0.00	0.05	ns
Rb	μg/L	Disease village	Non-disease village	0.53	0.97	0.08	ns
Mo	μg/L	Disease village	Non-disease village	1.41	0.42	0.21	ns
Cd	μg/L	Disease village	Non-disease village	0.01	0.01	0.17	ns
Cs	μg/L	Disease village	Non-disease village	0.00	0.01	0.05	*
Ba	μg/L	Disease village	Non-disease village	123.58	56.88	0.01	*
Pb	μg/L	Disease village	Non-disease village	0.04	0.18	0.01	**
U	μg/L	Disease village	Non-disease village	0.80	0.16	0.01	*

Note: “ns” non-significant; ***$P < 0.001$,**$P < 0.01$ and *$P < 0.05$ indicate that there are significant differences in the average element concentrations between the disease villages and non-disease villages

Table S8. The T-test results of the average element concentrations of soil in the KD villages in Shaanxi Province and SUD villages in Yunnan Province

Elements	Units	Processing 1	Processing 2	Mean (Processing 1)	Mean (Processing 2)	P value	Significance
Al	%	KD village	SUD village	6.53	4.46	0.01	*
Ca	%	KD village	SUD village	2.91	7.92	0.49	ns
Fe	%	KD village	SUD village	3.23	3.30	0.99	ns
K	%	KD village	SUD village	2.12	1.15	0.00	***
Mg	%	KD village	SUD village	1.13	1.25	0.66	ns
Na	%	KD village	SUD village	1.10	0.17	0.00	***
Se	mg/kg	KD village	SUD village	0.05	0.89	0.03	*
As	mg/kg	KD village	SUD village	20.51	82.92	0.35	ns
Mn	mg/kg	KD village	SUD village	655.83	607.85	0.27	ns
Ba	mg/kg	KD village	SUD village	479.38	209.60	0.00	***
Cr	mg/kg	KD village	SUD village	64.64	67.84	0.74	ns

Cu	mg/kg	KD village	SUD village	16.13	98.16	0.00	***
Li	mg/kg	KD village	SUD village	38.46	37.90	0.51	ns
Ni	mg/kg	KD village	SUD village	31.88	35.71	0.91	ns
Sr	mg/kg	KD village	SUD village	148.33	94.75	0.01	**
V	mg/kg	KD village	SUD village	86.72	103.70	0.02	*
Zn	mg/kg	KD village	SUD village	86.48	338.44	0.14	ns
Co	mg/kg	KD village	SUD village	13.83	15.18	0.98	ns
Ga	mg/kg	KD village	SUD village	37.35	22.51	0.00	**
Mo	mg/kg	KD village	SUD village	0.76	1.57	0.00	***
Cd	mg/kg	KD village	SUD village	0.29	3.21	0.06	ns
Cs	mg/kg	KD village	SUD village	7.35	8.50	0.89	ns
Pb	mg/kg	KD village	SUD village	14.97	124.24	0.02	*
U	mg/kg	KD village	SUD village	2.50	3.01	0.19	ns

Note: "ns" non-significant; ***$P < 0.001$,**$P < 0.01$ and *$P < 0.05$ indicate that there are significant differences in the average element concentrations between the KD villages in Shaanxi Province and SUD villages in Yunnan Province.

Table S9. The T-test results of the average element concentrations of corn in the KD villages in Shaanxi Province and SUD villages in Yunnan Province

Elements	Units	Processing 1	Processing 2	Mean (Processing 1)	Mean (Processing 2)	P value	Significance
Ca	mg/kg	KD village	SUD village	60.22	80.26	0.04	*
K	mg/kg	KD village	SUD village	2003.43	3275.50	0.00	**
Mg	mg/kg	KD village	SUD village	526.52	1236.00	0.00	***
Na	mg/kg	KD village	SUD village	20.37	32.64	0.00	***
P	mg/kg	KD village	SUD village	1530.68	2749.75	0.00	**
Al	mg/kg	KD village	SUD village	16.79	23.74	0.02	*
Ba	mg/kg	KD village	SUD village	0.23	0.14	0.19	ns
Cr	mg/kg	KD village	SUD village	0.51	0.66	0.03	*
Cu	mg/kg	KD village	SUD village	0.90	1.67	0.00	**

Fe	mg/kg	KD village	SUD village	21.78	16.33	0.36	ns
Mn	mg/kg	KD village	SUD village	2.76	4.25	0.02	*
Sr	mg/kg	KD village	SUD village	0.15	0.16	0.73	ns
Zn	mg/kg	KD village	SUD village	9.07	17.68	0.00	**
Co	mg/kg	KD village	SUD village	0.01	0.01	0.00	**
Se	µg/kg	KD village	SUD village	1.57	7.65	0.00	***
As	µg/kg	KD village	SUD village	8.13	0.90	0.01	*
Li	µg/kg	KD village	SUD village	17.03	11.72	0.38	ns
V	µg/kg	KD village	SUD village	13.10	2559.89	0.00	***
Ni	µg/kg	KD village	SUD village	134.60	162.84	0.83	ns
Ga	µg/kg	KD village	SUD village	240.91	8.97	0.00	***
Rb	µg/kg	KD village	SUD village	2435.03	4656.51	0.98	ns
Mo	µg/kg	KD village	SUD village	182.70	551.90	0.00	***
Cd	µg/kg	KD village	SUD village	9.66	4.29	0.80	ns
Cs	µg/kg	KD village	SUD village	15.67	35.45	0.46	ns
Pb	µg/kg	KD village	SUD village	79.49	43.78	0.00	***
U	µg/kg	KD village	SUD village	1.01	0.63	0.11	ns

Note: "ns" non-significant; ***$P < 0.001$,**$P < 0.01$ and *$P < 0.05$ indicate that there are significant differences in the average element concentrations between the KD villages in Shaanxi Province and SUD villages in Yunnan Province.

Table S10. The T-test results of the average element concentrations of drinking water in the KD villages in Shaanxi Province and SUD villages in Yunnan Province

Elements	Units	Processing 1	Processing 2	Mean (Processing 1)	Mean (Processing 2)	P value	Significance
Ca	mg/L	KD village	SUD village	86.01	102.12	0.92	ns
K	mg/L	KD village	SUD village	7.86	1.24	0.03	*
Mg	mg/L	KD village	SUD village	22.61	16.60	0.08	ns
Na	mg/L	KD village	SUD village	36.30	7.46	0.00	***
P	µg/L	KD village	SUD village	21.05	74.00	0.02	*

Al	μg/L	KD village	SUD village	0.00	0.40	0.05	ns
Fe	μg/L	KD village	SUD village	3.25	6.30	0.07	ns
Sr	μg/L	KD village	SUD village	631.05	1259.40	0.36	ns
Li	μg/L	KD village	SUD village	21.49	4.07	0.00	***
V	μg/L	KD village	SUD village	16.07	1.39	0.04	*
Cr	μg/L	KD village	SUD village	1.05	0.73	0.96	ns
Mn	μg/L	KD village	SUD village	2.75	1.38	0.87	ns
Co	μg/L	KD village	SUD village	0.25	0.04	0.00	***
Ni	μg/L	KD village	SUD village	1.05	0.85	0.41	ns
Cu	μg/L	KD village	SUD village	1.17	0.61	0.00	***
Zn	μg/L	KD village	SUD village	25.77	169.09	0.10	ns
Ga	μg/L	KD village	SUD village	5.23	0.01	0.00	***
As	μg/L	KD village	SUD village	2.86	0.71	0.00	***
Se	μg/L	KD village	SUD village	0.00	0.42	0.05	ns
Rb	μg/L	KD village	SUD village	20.21	0.53	0.00	**
Mo	μg/L	KD village	SUD village	9.29	1.41	0.11	ns
Cd	μg/L	KD village	SUD village	0.01	0.01	0.93	ns
Cs	μg/L	KD village	SUD village	0.10	0.00	0.02	*
Ba	μg/L	KD village	SUD village	154.58	123.58	0.18	ns
Pb	μg/L	KD village	SUD village	0.01	0.04	0.00	***
U	μg/L	KD village	SUD village	3.42	0.80	0.00	***

Note: "ns" non-significant; ***$P < 0.001$,**$P < 0.01$ and *$P < 0.05$ indicate that there are significant differences in the average element concentrations between the KD villages in Shaanxi Province and SUD villages in Yunnan Province.

Table S11. The statistical results of the element hazard quotient (HQ) in corn for residents of the disease villages in SUD area of Yunnan Province

**Items**	**Al**	**Ba**	**Cr**	**Cu**	**Fe**	**Mn**	**Sr**	**Zn**	**Co**	**Se**	**As**	**Li**	**Ni**	**Mo**	**Cd**	**Pb**	**U**

RFD (μg/kg/day)		1000	200	3	40	700	24	600	300	0.3	5	0.3	2	20	5	1	1.4	0.2
Adults	A'jiju	0.21	0.00	1.43	0.16	0.12	1.08	0.00	0.28	0.22	0.01	0.00	0.03	0.02	0.89	0.02	0.20	0.03
	Cangdi	0.15	0.00	1.12	0.33	0.17	1.52	0.00	0.38	0.10	0.01	0.00	0.01	0.02	0.56	0.02	0.09	0.01
	Huakou	0.12	0.00	1.95	0.29	0.15	1.04	0.00	0.42	0.22	0.02	0.08	0.04	0.05	0.94	0.05	0.36	0.03
	Xiaolongtan	0.16	0.00	1.31	0.32	0.19	1.05	0.00	0.49	0.22	0.01	0.00	0.07	0.14	0.54	0.03	0.18	0.02
Children	A'jiju	0.34	0.01	2.32	0.26	0.19	1.17	0.00	0.46	0.36	0.01	0.00	0.06	0.03	1.44	0.03	0.33	0.04
	Cangdi	0.25	0.01	1.82	0.54	0.27	1.65	0.00	0.62	0.17	0.01	0.00	0.02	0.02	0.92	0.03	0.14	0.02
	Huakou	0.19	0.01	3.17	0.47	0.25	1.13	0.00	0.68	0.36	0.03	0.13	0.06	0.07	1.53	0.08	0.58	0.04
	Xiaolongtan	0.25	0.01	2.13	0.53	0.30	1.14	0.00	0.79	0.36	0.01	0.00	0.11	0.22	0.88	0.04	0.30	0.03

Table S12. The statistical results of the element hazard quotient (HQ) in drinking water for residents of the disease villages in SUD area of Yunnan Province

**Items**		**Al**	**Ba**	**Cr**	**Cu**	**Fe**	**Mn**	**Sr**	**Zn**	**Co**	**Se**	**As**	**Li**	**Ni**	**Mo**	**Cd**	**Pb**	**U**
RFD (μg/L/day)		1000	200	3	40	700	24	600	300	0.3	5	0.3	2	20	5	1	1.4	0.2
Adults	A'jiju	0.00	0.01	0.01	0.00	0.00	0.00	0.10	0.00	0.00	0.00	0.06	0.07	0.00	0.02	0.00	0.00	0.19
	Cangdi	0.00	0.02	0.01	0.00	0.00	0.00	0.02	0.03	0.00	0.01	0.08	0.05	0.00	0.00	0.00	0.00	0.05
	Huakou	0.00	0.02	0.01	0.00	0.00	0.00	0.06	0.02	0.00	0.00	0.07	0.06	0.00	0.01	0.00	0.00	0.12
	Xiaolongtan	0.00	0.02	0.01	0.00	0.00	0.00	0.06	0.02	0.00	0.00	0.07	0.06	0.00	0.01	0.00	0.00	0.12
Children	A'jiju	0.00	0.03	0.02	0.00	0.00	0.00	0.26	0.00	0.01	0.00	0.14	0.17	0.00	0.04	0.00	0.00	0.45
	Cangdi	0.00	0.06	0.02	0.00	0.00	0.00	0.05	0.08	0.01	0.01	0.20	0.12	0.00	0.00	0.00	0.00	0.12
	Huakou	0.00	0.04	0.02	0.00	0.00	0.00	0.15	0.04	0.01	0.01	0.17	0.15	0.00	0.02	0.00	0.00	0.29
	Xiaolongtan	0.00	0.04	0.02	0.00	0.00	0.00	0.15	0.04	0.01	0.01	0.17	0.15	0.00	0.02	0.00	0.00	0.29

Table S13. The statistical results of the element hazard quotient (HQ) in corn for residents of the non-disease villages in SUD area of Yunnan Province

**Items**	**Al**	**Ba**	**Cr**	**Cu**	**Fe**	**Mn**	**Sr**	**Zn**	**Co**	**Se**	**As**	**Li**	**Ni**	**Mo**	**Cd**	**Pb**	**U**

RFD (μg/kg/day)		1000	200	3	40	700	24	600	300	0.3	5	0.3	2	20	5	1	1.4	0.2
Adults	Lishigeng	0.05	0.01	1.37	0.22	0.11	1.42	0.00	0.32	0.44	0.00	0.00	0.02	0.11	0.38	0.01	0.12	0.01
	Songping	0.10	0.01	1.36	0.30	0.12	1.25	0.00	0.49	1.46	0.00	0.07	0.02	0.09	0.24	0.01	0.12	0.01
	Gexi	0.01	0.00	0.98	0.37	0.16	0.90	0.00	0.38	0.89	0.02	0.12	0.03	0.07	0.90	0.13	0.17	0.01
	Zejiu	0.25	0.01	1.29	0.44	0.18	0.81	0.00	0.39	0.44	0.00	0.21	0.04	0.07	1.02	0.08	0.47	0.05
Children	Lishigeng	0.09	0.02	2.23	0.36	0.18	2.30	0.00	0.52	0.72	0.00	0.00	0.03	0.17	0.63	0.02	0.19	0.02
	Songping	0.16	0.01	2.20	0.49	0.20	2.04	0.00	0.79	2.38	0.00	0.11	0.04	0.15	0.39	0.02	0.20	0.02
	Gexi	0.03	0.00	1.59	0.61	0.26	1.46	0.00	0.61	1.44	0.04	0.19	0.05	0.11	1.47	0.21	0.28	0.02
	Zejiu	0.41	0.01	2.10	0.71	0.29	1.31	0.00	0.64	0.72	0.00	0.34	0.07	011	1.65	0.14	0.77	0.08

Table S14. The statistical results of the element hazard quotient (HQ) in drinking water for residents of the non-disease villages in SUD area of Yunnan Province

Items		Al	Ba	Cr	Cu	Fe	Mn	Sr	Zn	Co	Se	As	Li	Ni	Mo	Cd	Pb	U
RfD (μg/L/day)		1000	200	3	40	700	24	600	300	0.3	5	0.3	2	20	5	1	1.4	0.2
Adults	Lishigeng	0.00	0.01	0.01	0.00	0.00	0.00	0.04	0.28	0.01	0.00	0.05	0.09	0.00	0.00	0.00	0.00	0.05
	Songping	0.00	0.00	0.01	0.00	0.00	0.00	0.00	0.01	0.00	0.00	0.04	0.06	0.00	0.00	0.00	0.01	0.00
	Gexi	0.00	0.00	0.02	0.00	0.00	0.00	0.00	0.00	0.00	0.01	0.01	0.00	0.00	0.00	0.00	0.00	0.13
	Zejiu	0.00	0.00	0.01	0.00	0.00	0.00	0.01	0.00	0.00	0.00	0.03	0.01	0.00	0.01	0.00	0.00	0.07
Children	Lishigeng	0.00	0.03	0.02	0.00	0.00	0.01	0.09	0.67	0.01	0.00	0.12	0.21	0.00	0.01	0.00	0.00	0.11
	Songping	0.00	0.01	0.02	0.00	0.00	0.01	0.01	0.02	0.00	0.00	0.10	0.15	0.00	0.00	0.00	0.01	0.00
	Gexi	0.00	0.00	0.04	0.00	0.00	0.00	0.01	0.00	0.01	0.02	0.04	0.01	0.01	0.00	0.00	0.00	0.31
	Zejiu	0.00	0.01	0.02	0.00	0.00	0.00	0.02	0.01	0.01	0.00	0.08	0.02	0.01	0.02	0.00	0.00	0.16
